# Systematic Targeting of GD2‐Positive Neuroblastoma Tumors With a Photooncolytic Phage Nanovector Platform

**DOI:** 10.1002/advs.202415356

**Published:** 2025-08-13

**Authors:** Suleman Khan Zadran, Nicola Facchinello, Piergiuseppe De Rosa, Roberto Saporetti, Paolo Emidio Costantini, Luca Ulfo, Michela Nigro, Annapaola Petrosino, Lucia Pappagallo, Sara Aloisi, Giorgio Milazzo, Zainul Abe Din, Alberto Rigamonti, Leonardo Flora, Martina Santulli, Leonardo Cimadom, Giampaolo Zuccheri, Mattia Zangoli, Manuele Di Sante, Matteo Di Giosia, Francesca Di Maria, Roberto Bernardoni, Eveline Barbieri, Matteo Calvaresi, Alberto Danielli, Giovanni Perini

**Affiliations:** ^1^ Department of Pharmacy and Biotechnology Alma Mater Studiorum – University of Bologna via Francesco Selmi 3 Bologna 40126 Italy; ^2^ Department of Chemistry “Giacomo Ciamician” Alma Mater Studiorum – University of Bologna Via Francesco Selmi 2 Bologna 40126 Italy; ^3^ Institute for Organic Synthesis and Photoreactivity (ISOF) National Research Council of Italy (CNR) Via P. Gobetti 101 Bologna I‐40129 Italy; ^4^ Istituto di Ricerca e Cura a Carattere Scientifico (IRCCS) AOUBO Sant Orsola – Laboratory of Preclinical and Translational Research in Oncology (PRO) Bologna 40138 Italy; ^5^ Department of Pediatrics Section of Hematology‐Oncology Texas Children's Cancer and Hematology Centers Baylor College of Medicine Houston TX 77030 USA; ^6^ Istituto di Ricerca e Cura a Carattere Scientifico (IRCCS) Istituto delle Scienze Neurologiche di Bologna Programma di Neurogenetica Bologna 40139 Italy

**Keywords:** CRISPRa, M13 bacteriophage biotherapeutics, neuroblastoma GD2, photodynamic therapy, zebrafish xenograft

## Abstract

Disialoganglioside‐GD2 is a key molecular target for Neuroblastoma (NB) immunotherapy based on the employment of GD2‐targeting antibodies. However, about 50% of treated patients can experience tumor relapse due to limited immune‐mediated cytotoxicity and poor antibody penetration into tumors. To address this problem, a tumor‐penetrating photo‐oncolytic phage nanovector platform is genetically and chemically developed that selectively targets GD2‐expressing NB cells. The phage bioconjugates, functionalized with different photosensitizers, result in specific and selective oncolysis of GD2‐positive NB cells upon light irradiation, without affecting GD2‐negative ones. The photo‐oncolytic phage vectors are shown to deeply penetrate into GD2‐positive tumor spheroids in vitro, and to cross biological barriers in a zebrafish xenograft model, maintaining their ablation specificity upon irradiation. Finally, to overcome resistance from GD2 loss, often linked to poor prognosis, a CRISPRa strategy is introduced to reactivate GD2 expression in GD2‐negative cells. The approach offers a minimally invasive and highly effective strategy, addressing unmet needs in NB therapy.

## Graphical abstract

A tumor‐penetrating photooncolytic phage is developed for selectively targeting and delivering photosensitizers to GD2‐positive Neuroblastoma cells. Upon light activation, it precisely induces oncolysis of GD2‐positive cells with no effect on GD2‐negative ones. It penetrates deep into tumors and kills cells in zebrafish xenograft models, offering a precise and minimally invasive treatment approach.

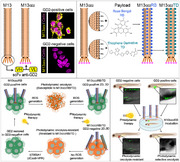

## 1 Introduction

Neuroblastoma (NB) is one of the most common pediatric neuroendocrine tumors originating from neural crest‐derived sympathoadrenal precursor cells and accounts for 15% of total pediatric cancer mortalities.^[^ ^1^ ^]^ NB is a highly heterogeneous disease ranging from asymptomatic malignancy with spontaneous regression to aggressive metastatic disease, culminating in poor clinical outcome.^[^ ^2^ ^]^ Recent treatments for High‐Risk NB rely on synergistic approaches consisting of conventional chemotherapy,^[^ ^3^ ^]^ radiation,^[^ ^4^ ^]^ surgical resections,^[^ ^5^ ^]^ and anti‐GD2 monoclonal antibodies.^[^ ^6^ ^]^ Despite rigorous treatment strategies, the overall 5‐year survival rates remain below 50% and the implemented medications exhibit substantial late adverse effects.^[^ ^7^ ^]^

In response to these challenges, there has been a concerted effort in oncology to adopt synergistic treatment approaches, aiming at improving therapeutic outcomes and mitigating treatment‐related toxicity.^[^ ^8^ ^]^ One such promising avenue is photodynamic therapy (PDT),^[^ ^9^ ^]^ an FDA‐approved modality that harnesses the synergistic interplay among a photosensitizer (PS), light, and oxygen to directly induce tumor cell death,^[^ ^10^ ^]^ vascular destruction,^[^ ^11^ ^]^ or immune system activation against tumors.^[^ ^12^ ^]^ Interestingly, the intrinsic fluorescence feature of the PS can enable the diagnosis of early malignancies, precancerous lesions, tumor margins, and dysplastic tissues after surgical resection of tumors.^[^ ^13^ ^]^ Notwithstanding that, the clinical efficacy of PDT is limited by the accumulation of the PS outside the tumor lesion, causing organ toxicity.^[^ ^14^ ^]^ One way to overcome this problem is to deliver the PS to the tumor lesion by targeting surface molecules (e.g., transmembrane receptors, glycolipids, etc.) that are overexpressed in the tumor cell.^[^ ^15^ ^]^

In NB, one such surface molecule is GD2, a disialoganglioside that is highly expressed in most NB cells (5‐10 million molecules/cell) of the patients, whereas it is almost absent in tissues of healthy individuals.^[^ ^16^ ^]^ Poor prognosis of NB patients and rapid progression are also associated with GD2 expression.^[^ ^17^ ^]^ The specific role of GD2 is still unclear, although it is hypothesized that it enhances tumor cell proliferation,^[^ ^18^ ^]^ cell‐to‐cell adhesion,^[^ ^19^ ^]^ motility, invasion, and migration.^[^ ^20^ ^]^ Of interest, based on the level of expression, therapeutic effect, immunogenicity, and level of antigen specificity, the U.S. National Institutes of Health ranked GD2 twelfth among 75 potential molecular targets.^[^ ^21^ ^]^ GD2‐expressing tumors have been targeted with multi‐modal therapies by using an FDA‐approved anti‐GD2 monoclonal antibody (dinutuximab) used in combination therapy for high‐risk neuroblastoma.^[^ ^22^ ^]^ Despite the fact that anti‐GD2 therapy has positively impacted survival of children with high‐risk NB by 20%,^[^ ^22b^ ^]^ nearly half of the patients treated with the monoclonal antibody experience recurrence.^[^ ^8a^ ^]^ However, some NB tumors become resistant to anti‐GD2 therapy, and the immunosuppressive nature of NB can impede the effectiveness of the therapy by modulating the response to treatment, making it harder for the immune system to target and eliminate NB cells effectively.^[^ ^23^ ^]^ This resistance mechanism poses a significant challenge in achieving successful treatment outcomes for patients with NB who either do not respond to or develop resistance to the anti‐GD2 immunotherapy.^[^ ^24^ ^]^ To address these problems, we adopted a novel approach that employs the M13 filamentous phage as a nanobiotechnological and photo‐oncolytic tool for targeted therapy, leveraging on the proven physical and oxidative effects triggered by the photodynamic activation of the PS‐rigged phage vectors.^[^ ^25^ ^]^ M13 phage offers a versatile platform for integrating molecular targeting and photochemistry. Compared to other targeted delivery systems, it provides several advantages: ease of genetic manipulation of the vector platform, lack of replication in eukaryotic cells, enhanced targeting affinity deriving from cooperative binding via pentavalent scFv display on the pIII minor coat protein, cost‐effective GMP production processes, innate capability to penetrate tissues and biological barriers, and the potential for high PS loading through the multiple conjugation sites available on the major coat protein pVIII.^[^ ^25^, ^26^ ^]^ Notably, M13 has already been successfully employed in phage‐based photodynamic therapy against cancer cells overexpressing either EGFR or integrin α5β1.^[^ ^26^, ^27^ ^]^

Specifically, the M13 phage was genetically engineered to express the single‐chain variable fragment (scFv) derived from the human 14.G2a anti‐GD2 monoclonal antibody (approved by the FDA and EMA for immunotherapy) at the tip of the pIII phage protein. This allows precise targeting of the phage to cells expressing only GD2.

**Scheme 1:**
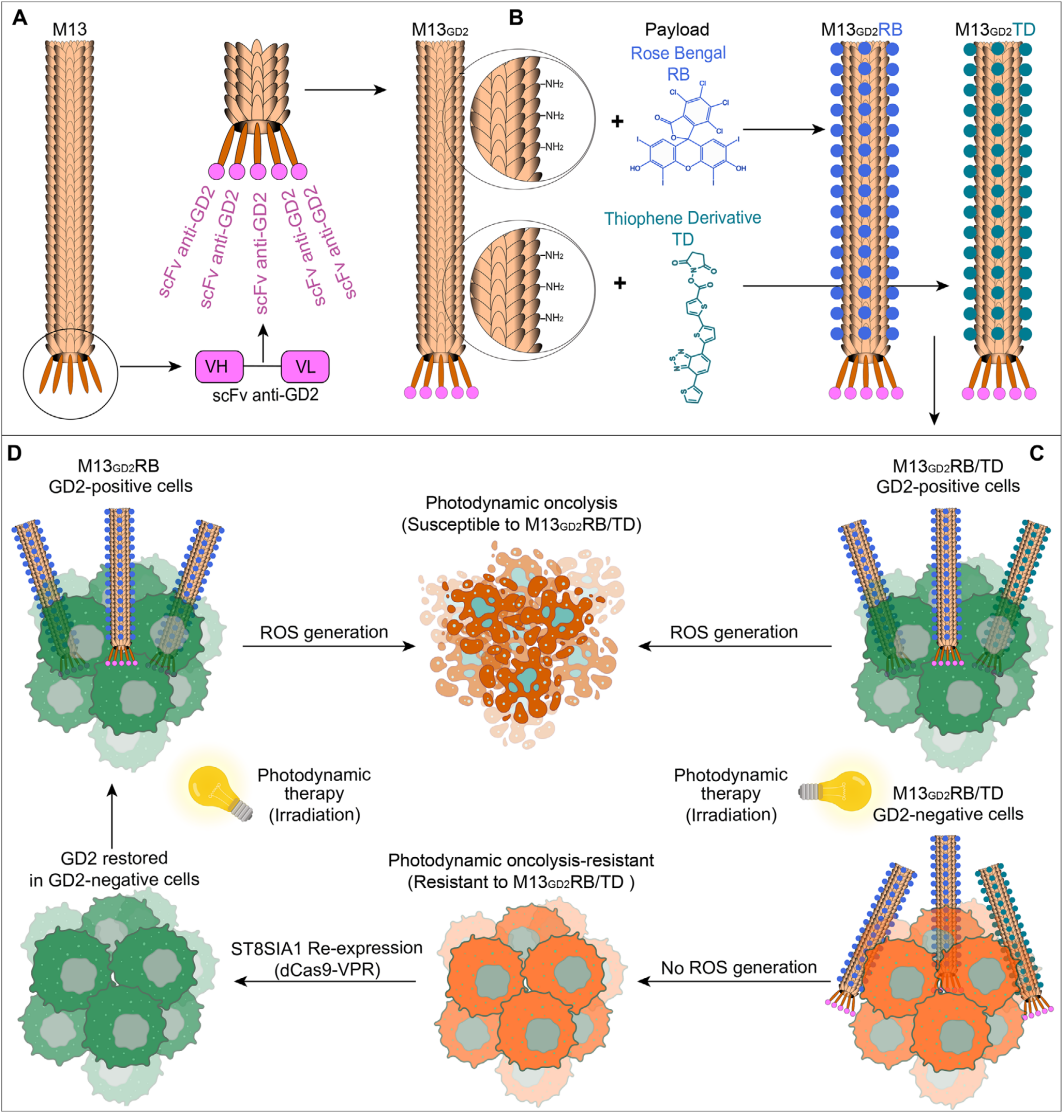
Orthogonal engineering of M13 phage for selectively targeting and eliminating GD2‐positive cells. **A)** Genetic modification of M13 pIII fused with scFv (14G2a) retargeting GD2 (M13_GD2_). **B)** Chemical functionalization of M13_GD2_ with RB and TD, generating M13_GD2_RB or M13_GD2_TD, respectively. **C)** Schematic representation of PDT carried out with M13_GD2_RB or M13_GD2_TD on GD2‐positive and GD2‐negative cells induces selective cytotoxicity in GD2‐positive cells. **D)** Restoration of GD2 in GD2‐negative NB cells (anti‐GD2 resistant) and M13_GD2_RB‐mediated PDT.

Our results show that the phages loaded with Rose Bengal (RB) or thiophene derivative (TD) sensitizers can target and selectively kill GD2‐positive and dinutuximab‐resistant NB cells upon exposure to white LED light irradiation (**Scheme** **1**). Moreover, we employed conditional activation of the ST8SIA1 gene through CRISPRa technology to re‐sensitize GD2‐negative NB cells to the photo‐oncolytic effect mediated by the phage nanovector.

## 2 Results

### 2.1 Dissection of the Metabolic Pathway Underpinning Differential GD2 Biosynthesis in NB Cell Lines

**Figure 1:**
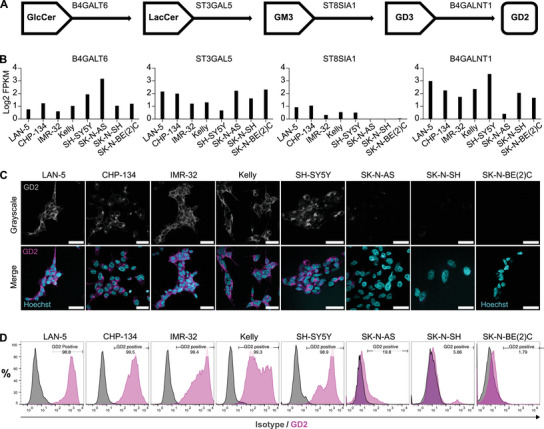
Characterization of GD2 expression in NB cell lines. A) Enzymes involved in the biosynthesis pathway of GD2. **B)** Gene expression of key enzymes involved in the GD2 biosynthesis pathway in a panel of NB cell lines. B4GALT6, ST3GAL5, and B4GALNT1 are highly expressed in all panels of NB cell lines, while ST8SIA1, which converts GM3 into GD3, is poorly expressed in SK‐N‐AS, SK‐N‐SH, and SK‐N‐BE(2)C. **C)** Immunofluorescence staining of GD2 in a panel of NB cell lines. GD2 was stained with mouse mAb anti‐GD2, followed by a secondary anti‐mouse FITC‐conjugated antibody (magenta), and nuclei were stained with Hoechst 33342 (cyan). All scale bars represent 30 µm. **D)** Flow cytometry analysis with anti‐GD2 antibody followed by FITC‐conjugate anti‐mouse IgG. Magenta peaks indicate the GD2 expression, while black peaks represent cells stained with isotype control IgG.

Given the different levels of GD2 expression reported in various NB cellular models,^[^ ^28^ ^]^ we investigated the expression of key genes involved in the GD2 biosynthetic pathway in a panel of NB cell lines. The pathway for GD2 biosynthesis requires four essential enzymes, B4GALT6, ST3GAL5, ST8SIA1, and B4GALNT1,^[^ ^29^ ^]^ which sequentially convert glucosylceramide (GlcCer) into lactosylceramide (LacCer), GM3, GD3, and finally into GD2, respectively (**Figure** **1**A). Among these enzymes, ST8SIA1 catalyzes the conversion of GM3 into GD3, whereas B4GALNT1 directly catalyzes GD2 synthesis from the GD3 precursor. According to the RNAseq analysis (R2 database, Maris dataset, *n* = 41) of a reference panel of NB lines, it is shown that B4GALT6, ST3GAL5, and B4GALNT1 are consistently expressed across the entire panel of cell lines, whereas *ST8SIA1*, encoding the enzyme that converts GM3 into GD3, is expressed at low level or not at all in SK‐N‐AS, SK‐N‐SH and SK‐N‐BE(2)C NB cell lines (Figure 1B **)**. Consistent results regarding the *ST8SIA1* mRNA were also observed in our cell lines (Figure S1A, Supporting Information). Further western blotting also demonstrated that ST8SIA1 expression, at the protein level, is absent in SK‐N‐AS, SK‐N‐SH, and SK‐N‐BE(2)C NB cell lines (Figure S1B, Supporting Information). To correlate ST8SIA1 expression with GD2 biosynthesis and expression, immunofluorescence (IF) assays were carried out to confirm the lack of GD2 staining in SK‐N‐AS, SK‐N‐SH, and SK‐N‐BE(2)C (Figure 1C). Flow cytometry (FC) was applied to further corroborate the variability of GD2 expression in each cell line. LAN‐5 and CHP‐134 displayed a strong and homogeneous expression of GD2, while IMR‐32, Kelly, and SH‐SY5Y showed a more heterogeneous expression pattern. Conversely, a small percentage of SK‐N‐AS cells exhibited weak positivity for GD2, whereas SK‐N‐SH and SK‐N‐BE(2)C were essentially negative (Figure 1D).

### 2.2 Retargeting of the M13 Phage to GD2‐Expressing NB Cells

**Figure 2:**
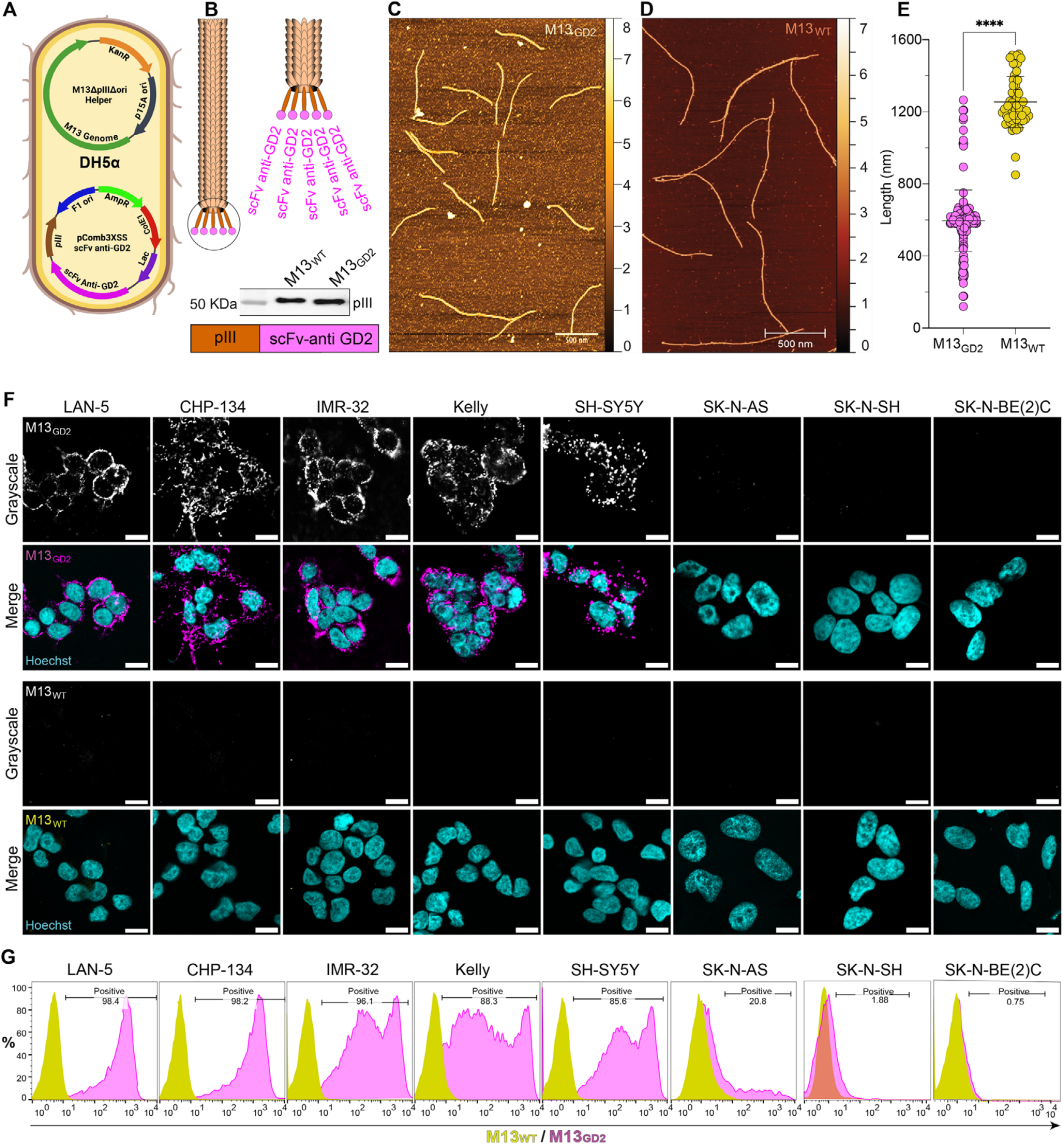
M13_GD2_ engineering and validation. A) Schematic representation of the M13_GD2_ generation (see experimental procedures for details). **B)** Immunoblot validating display of scFv‐pIII display (43 kDa) on M13_GD2_, compared to wild‐type pIII (42.5 kDa) on M13_WT_. **C)** AFM micrograph of purified M13_GD2_ vector. **D)** AFM micrograph of purified M13_WT_. **E)** Size distribution of M13_GD2_ and M13_WT_ determined by AFM, each single dot represents a phage, M13_GD2_ mean length 600 nm ± 54.6 nm and M13_WT_ mean length 1254 nm ± 141.8 nm, respectively, *p* = 0.000. **F)** Validation of targeting and specificity of M13_GD2_ and M13_WT_ on a panel of NB cell lines, M13_GD2_ indicating strong binding to GD2‐positive cells, whereas minimal or no binding to GD2‐negative cells by M13_GD2_ and M13_WT_. Immunostaining: pVIII of M13_GD2_ (magenta), pVIII of M13_WT_ (yellow), nuclei (Hoechst, cyan). All scale bars represent 10 µm. **G)** Flow cytometry validating the binding specificity of M13_GD2_ and M13_WT_ on a panel of NB cell lines by immunostaining: pVIII of M13_GD2_ (magenta peaks), pVIII of M13_WT_ (yellow peaks).

To generate a GD2‐targeting vector, we leveraged an anti‐GD2 single‐chain variable fragment (scFv) derived from 14.G2a, a therapeutic monoclonal antibody approved for NB immunotherapy by the FDA and EMA.^[^ ^30^ ^]^ A codon‐optimized cistron for this GD2‐specific scFv was fused to the C‐terminal domain of the minor coat protein pIII, displayed in five copies at the tip of the M13 phage. Co‐transformation of *E. coli* DH5α with a phagemid carrying the scFv‐pIII fusion under the control of an isopropyl ß‐D‐1‐thiogalactopyranoside (IPTG)‐inducible promoter and a helper plasmid providing all the necessary M13 genes except for pIII and the f1 origin, allowed the production of a phage with the pentavalent display of the anti‐GD2 scFv (**Figure** **2**A). The correct expression of the fusion was validated by immunoblotting, and a band of the expected molecular weight (43 kDa) was observed using an anti‐M13 pIII antibody, thus confirming the incorporation of modified pIII into the M13_GD2_ phage particles (Figure 2B). Next, the morphology and structural integrity of the M13_GD2_ phages were evaluated through atomic force microscopy (AFM) (Figure 2C) and compared to the wild‐type M13 virions (M13_WT_) (Figure 2D). The M13_GD2_ virions maintained an intact architecture without substantial structural abnormalities, though presenting a shortened length as compared to the M13_WT_, consistent with the packaging of the shorter 4065‐bp phagemid DNA (instead of the modified helper phage DNA lacking the f1 origin) (Figure 2E). After optimization of the concentrations required to monitor the phage binding to NB cells by using an anti‐pVIII antibody to detect the M13 virions bound to NB cells (Figure S2A, Supporting Information), the targeting efficacy of M13_GD2_ was monitored in IF assays against the same panel of NB cells characterized for GD2 expression (Figure 1C,D) and M13_WT_ phages were used as a negative control. Consistently, M13_GD2_ binding was observed only for GD2‐positive cell lines, while M13_WT_ virions failed to bind to either GD2‐positive or GD2‐negative cells (Figure 2F), as also validated by the quantification of the Mean Fluorescence Intensity (MFI) of the pVIII signal (Figure S2B, Supporting Information). A non‐NB cell line (HEK293) was also included for validation and co‐cultured with GD2‐positive LAN‐5 cells, further validating the specificity of M13_GD2_ in a mixed population of GD2‐positive and GD2‐negative cells (Figure S1C, Supporting Information). The specific tropism of M13_GD2_ for GD2‐positive cells was also corroborated in FC, using a FITC‐conjugated anti‐pVIII antibody that fluorescently labels the phages. Results show that levels of bound M13_GD2_ strongly correlate with the levels of GD2 expression in the NB cells (Figure 2G). Consistently, the M13_GD2_ phages, directly conjugated with hundreds of CF488 fluorophores per virion, maintained their specificity toward GD2‐positive cells in FC (Figure S2C, Supporting Information). These results demonstrate the specificity and robustness of M13_GD2_ as a GD2‐targeting vector.

### 2.3 M13_GD2‐_Rose Bengal (M13_GD2_RB) Bioconjugate Selectively Binds and Kills GD2‐expressing Cells

**Figure 3:**
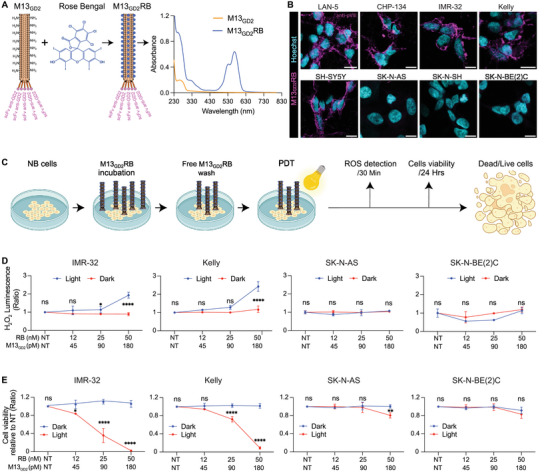
Conjugation of M13GD2 with RB, targeting validation and specific photosensitization of GD2‐expressing NB cells by M13_GD2_RB bioconjugates. A) Functionalization of M13_GD2_ with RB. UV–vis absorption spectrum of M13_GD2_ (orange line) and RB‐conjugated phages M13_GD2_RB (royal blue line). **B)** Validation of M13_GD2_RB tropism and specificity by IF microscopy: pVIII of M13_GD2_RB (magenta) and nuclei (cyan). All scale bars represent 10 µm. **C)** Schematic representation of the PDT experiment. **D)** ROS generation (determined by ROS‐Glo H_2_O_2_ Assay) on GD2‐positive (IMR‐32 and Kelly) and GD2‐negative cell lines (SK‐N‐AS and SK‐N‐BE(2)C) irradiated with white LED light (blue) or control kept in the dark (red). **E)** Survival rates determined by MTT assay of GD2‐positive and GD2‐negative cell lines after M13_GD2_RB‐mediated PDT; irradiated sample (red) (24 mW cm^−^^2^ irradiance), dark controls (blue). Data are presented as the mean ± SD of 3 experiments. Statistical significance was determined using a two‐way ANOVA followed by Šídák's multiple comparisons test. The significance of differences between light and dark conditions is indicated as ns (not significant, *p* > 0.05), ^*^ (*p* < 0.05), ^**^(*p* < 0.01), ^***^(*p* < 0.001), ^****^ (*p* < 0.0001).

Next, to test M13_GD2_ as a potential therapeutic vector, we functionalized its capsid by conjugation with Rose Bengal (M13_GD2_RB). Rose Bengal is a well‐characterized xanthene dye known for its high quantum yield in generating reactive oxygen species (ROS) under visible light (500–600 nm). It is commercially available, cost‐effective, biocompatible, and it holds FDA orphan drug status for ocular diagnostics,^[^ ^31^ ^]^ several malignancies^[^ ^25^, ^32^ ^]^ and antimicrobial applications^[^ ^25^, ^33^ ^]^ in PDT approaches. After purification of the M13_GD2_RB phage, UV–vis absorption spectroscopy was used to infer phage concentration and estimate the payload of sensitizers (228 ± 50, RB molecules/phage) (**Table** **1**) (**Figure** **3**A **)**. To exclude the possibility that the high number of sensitizers could impinge upon the phage tropism and specificity, M13_GD2_RB bioconjugates were tested in IF for their binding to NB cells. Results show consistent binding of M13_GD2_RB to LAN‐5, CHP‐134, IMR‐32, Kelly, and SH‐SY5Y (GD2‐positive), while interaction with GD2‐low and GD‐negative lines (respectively SK‐N‐AS, SK‐N‐SH, and SK‐N‐BE(2)C) proved undetectable, demonstrating preservation of the M13_GD2_RB vector tropism towards GD2‐expressing cells (Figure 3B).

Consequently, we determined the photodynamic activity of M13_GD2_RB on the two GD2‐positive cell lines (IMR‐32 and Kelly), including SK‐N‐AS and SK‐N‐BE(2)C as negative controls. Cells were incubated with various concentrations of M13_GD2_RB (45, 90, and 180 pM phage vector, corresponding to 12, 25, and 50 nM RB equivalents, respectively). Following the removal of free phages by washing, cells were irradiated with a low‐power white LED light (24 mW cm^−^^2^ irradiance), whereas non‐irradiated controls were kept in the dark (Figure 3C). Light‐activation of photosensitizer is known to trigger the production of cytotoxic reactive oxygen species (ROS).^[^ ^34^ ^]^ A significant dose and light‐dependent increase in the production of ROS was recorded exclusively for GD2‐positive cells. In contrast, ROS levels remained comparable in GD2‐negative cells (Figure 3D). Moreover, upon irradiation, an increase in intracellular ROS was observed in GD2‐positive cells but not in GD2‐negative ones (Figure S3A, Supporting Information), indicating a selective delivery of photoactivatable photosensitizers by M13_GD2_. Finally, survival rates of treated cells were assessed with a metabolic assay (MTT) 24 h post‐irradiation. Compared to controls kept in the dark, significant photosensitization levels were observed in IMR‐32 and Kelly cells in a dose‐dependent manner, starting from picomolar concentrations of the phage vector. Remarkably, no significant effects were observed in the GD2‐negative lines, even at the highest phage concentration tested, with the exception of SK‐N‐AS cells (Figure 3E), in which a small population (around 19%) expresses a low level of GD2 (Figure 1C,D; Figure S1A, Supporting Information). Likewise, no significant dark toxicity was observed in either GD2‐positive or GD2‐negative cells in the absence of irradiation (Figure 3E). These results demonstrate that M13_GD2_RB mediates specific and light‐triggerable killing of GD2‐expressing NB cells. Annexin‐V and propidium iodide (PI) labeling of cells was carried out to further explore the mechanisms of photodynamic cell death triggered by M13_GD2_RB. Results indicated the predominance of necrotic cell death for GD2‐positive cell lines upon irradiation, whereas no or minimal staining for Annexin‐V and PI was observed for GD2‐negative cells (Figure S3B, Supporting Information).

### 2.4 Re‐Expression of GD2 Sensitizes GD2‐Negative NB Cells to M13_GD2_RB‐Mediated PDT

**Figure 4:**
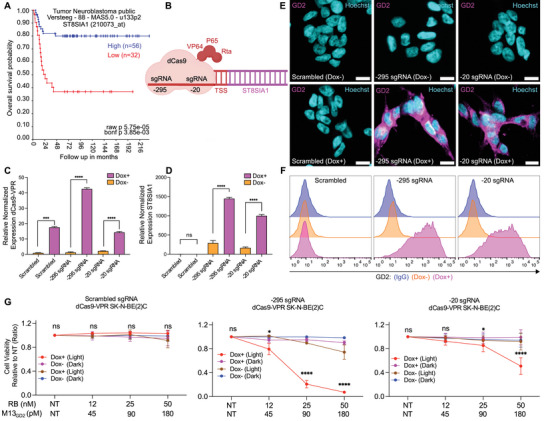
Re‐activation of GD2 expression sensitizes SK‐N‐BE(2)C cells to M13_GD2_RB‐mediated PDT. **A)** Low ST8SIA1 expression correlates with poor prognosis in NB patients using the NB cohort (Versteeg dataset, *n* = 88). **B)** Representation of the sgRNAs dCas9‐VPR complex. The dCas9 is directly linked at its C‐terminal end to transcription activators, namely VP64, P65, and Rta (VPR). The sgRNAs were designed to target regions upstream of the ST8SIA1 gene's transcription start site (TSS) for transcription activation. The sgRNAs pinpoint specific sites within the proximal promoter region of ST8SIA1. The numbering of the sgRNAs corresponds to their distance in base pairs (bp) from the TSS of ST8SIA1 mRNA transcript variant 1. **C)** Relative normalized expression of dCas9‐VPR by Doxycycline (Dox) inducible Tet‐On transactivator (rtTA) in SK‐N‐BE(2)C cell line, scrambled sgRNA used as a control. **D)** Relative normalized expression of ST8SIA1 mRNA in qRT‐PCR in SK‐N‐BE(2)C. **E)** Immunostaining of GD2 in ± Dox condition (GD2: magenta), (nuclei: cyan). **F)** Flow cytometry of GD2 by anti‐GD2 antibody followed by secondary FITC‐conjugated mouse IgG. Blue bars show the isotype control IgG, Orange peaks show Dox‐ conditions of GD2 expression, while magenta peaks show Dox+ conditions of GD2 expression. **G)** Survival rates determined by MTT assay of scrambled sgRNA, −295 sgRNA and −20 sgRNA (± Dox) in irradiated (Light) (24 mW cm^−^^2^ irradiance) and non‐irradiated (Dark) conditions after 24 h M13_GD2_RB‐mediated PDT treatment. Data are presented as the mean ± SD of 4 experiments. Statistical significance was calculated by two‐way ANOVA followed by Šídák's multiple comparisons test. The significance of differences between light and dark conditions is indicated as ns (not significant, *p* > 0.05), ^*^(*p* < 0.05), ^**^(*p* < 0.01), ^***^(*p* < 0.001), ^****^ (*p* < 0.0001).

Anti‐GD2 antibodies have revolutionized neuroblastoma treatment, increasing survival rates by up to 20%.^[^ ^6e^ ^]^ Despite this progress, GD2‐negative tumor cells still resist GD2‐directed immunotherapies. Indeed, we found a significant correlation between low ST8SIA1 expression and poor prognosis in NB patients using the NB cohort (Versteeg dataset, *n* = 88) (**Figure** **4**A). Owing to the central role of ST8SIA1 in the biosynthesis of GD2 (Figure 1), we surmised that reactivation of ST8SIA1 could restore susceptibility to GD2‐targeting therapies, including M13_GD2_RB. From a panel of NB cell lines, we strategically selected the SK‐N‐BE(2)C cell line, characterized by minimal ST8SIA1 expression and absence of GD2 (Figure 1B,D), as an ideal model for reactivating ST8SIA1 transcription. To achieve targeted ST8SIA1gene activation we employed a doxycycline‐inducible dCas9‐VPR transactivation system. After stable integration of dCas9‐VPR into SK‐N‐BE(2)C, cells were subsequently transduced with lentiviral constructs expressing two specific sgRNAs designed to target positions at −295 bp and −20 bp upstream of the ST8SIA1 transcription start site (TSS) (Figure 4B). The expression of dCas9‐VPR significantly increased following doxycycline incubation (Figure 4C), corresponding to the significant activation of ST8SIA1 by both sgRNAs, whereas no significant activation was observed with a scrambled sgRNA controls (Figure 4D). Following the re‐expression of ST8SIA1, we carried out IF and FC to determine whether GD2 expression was reinstated. Cells stained positive for GD2 upon ST8SIA1 dCas9‐VPR induction (Figure 4E), and ≈60% (−20 sgRNA) or 85% (−295 sgRNA) became positive for GD2 in FC (Figure 4F). No reactivation of GD2 expression was observed with the scrambled sgRNA controls (Figure 4E,F). This indicates that GD2 expression can be restored through activation of the transcription of ST8SIA1 and demonstrates that the re‐expression of this gene is sufficient for GD2 biosynthesis, rendering these cells potentially vulnerable to GD2‐directed therapies. Consistently, the effect of reactivated GD2‐expression on M13_GD2_RB‐mediated photosensitization in dCas9‐VPR‐SK‐N‐BE(2)C cells was evaluated (Figure 4E,F). Compared to uninduced controls, dox‐induced dCas9‐VPR‐expressing SK‐N‐BE(2)C cells showed significant photosensitization by M13_GD2_RB (achieving approximatly 50% and 90% cytotoxicity in the −20 sgRNA and −295 sgRNA groups, respectively), whereas no significant effects were observed when cells were kept in the dark or transduced with a scrambled sgRNA and independently from the presence of the inducer (Figure 4G). These findings highlight the potential therapeutic implications of GD2 re‐activation and demonstrate that M13_GD2_ phages loaded with RB are specific and efficient vectors for the photoablation of GD2‐positive cells.

To further elucidate the capability of M13_GD2_RB to selectively target and eliminate GD2‐positive cells within a mixed population of both GD2‐positive and GD2‐negative cells, we stably transduced Kelly (GD2‐positive) and SK‐N‐AS (GD2‐negative) cells to express either *Renilla reniformis* or firefly luciferase, respectively. The use of Renilla luciferase (480 nm) and firefly luciferase (560 nm), which emit at distinct, non‐overlapping wavelengths, allows for independent and simultaneous monitoring of each cell population in co‐culture. Following M13_GD2_RB‐mediated PDT, luminescence intensity is directly proportional to the number of viable cells, enabling differential cytotoxicity assessment (Figure S4A, Supporting Information). Co‐cultured cells were incubated with various concentrations of M13_GD2_RB and subsequently subjected to irradiation. Controls were kept in the dark, and luciferase activity was measured 24 h post‐irradiation. Results showed significant photoablation for Kelly cells, whereas SK‐N‐AS cells displayed remarkably higher survival rates (Figure S4B, Supporting Information**)**. Nonetheless, a slight decrease in survival could also be observed for the co‐cultured SK‐N‐AS cells, as compared to SK‐N‐AS monocultures. This effect is likely due to a small subpopulation of cells (around 19%) with low‐level GD2 expression (Figure 1), or the spreading of extracellular ROS from GD2‐positive cells to adjacent GD2‐negative ones, or it could be a minor bystander effect.^[^ ^35^ ^]^

### 2.5 The M13_GD2_ Vector Mediates the Targeted Delivery of an Oligothiophene‐Based Photosensitizer for the Photodynamic Killing of GD2‐Positive Cells

**Figure 5:**
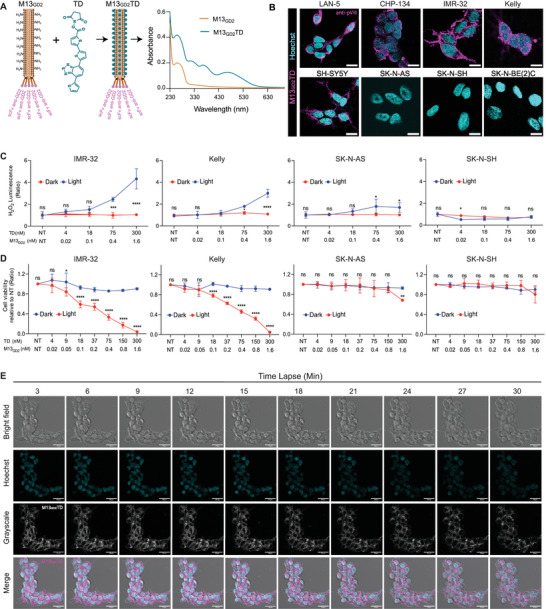
Conjugation of M13_GD2_ with TD, retargeting, Photodynamic killing, and Real‐time monitoring. A) M13_GD2_ pVIII functionalization with TD. UV–vis absorbance spectrum: orange line represents the nude phage (M13_GD2_)_,_ while the dark cyan line represents the TD‐conjugated phages (M13_GD2_TD). **B)** The conjugated phages were tested on a panel of cell lines for targeting specificity. Immunostaining: pVIII of M13_GD2_TD (magenta) and the nuclei (cyan). All scale bars represent 10 µm. **C)** ROS production was determined by ROS Glo assay in GD2‐positive or GD2‐negative cell lines irradiated with white LED light after 1 h post‐PDT treatment with M13_GD2_TD, controls were kept in the dark. **D)** Survival rates were assessed by MTT assay of GD2‐positive and GD2‐negative cell lines, irradiated with a white light‐LED light (24 mW cm^−^^2^ irradiance) after 24 h post‐PDT treatment with M13_GD2_TD, controls were kept in the dark. Data presents the mean ± SD of 3 experiments. **E)** Real‐time monitoring of events occurring in IMR‐32 upon M13_GD2_TD‐mediated PDT. Cells were observed using an excitation wavelength of 561 nm and an emission wavelength of 550–650 nm: M13_GD2_TD (magenta), nuclei (cyan). Statistical significance was calculated by two‐way ANOVA followed by Šídák's multiple comparisons test. The significance of differences between light and dark conditions is indicated as ns (not significant, *p* > 0.05), ^*^ (*p* < 0.05), ^**^(*p* < 0.01), ^***^(*p* < 0.001), ^****^ (*p* < 0.0001).

To demonstrate the general validity of our modular approach, the phage vector platform was functionalized with a different photosensitizer, such as an oligothiophene derivative (TD), resulting in a high‐performing phototheranostic agent.^[^ ^25^, ^36^ ^]^ The thiophene derivative, synthesized by our research laboratories, was chosen for its multifunctional phototheranostic profile.^[^ ^26^, ^37^ ^]^ TD exhibits balanced fluorescence, ROS generation, and photostability, with tunable optical properties. Thiophene‐based compounds have demonstrated utility across photodynamic, photothermal, and sonodynamic therapies,^[^ ^38^ ^]^ and their robust phototransduction capabilities enable bioimaging (i.e., fluorescence, photoacoustic) and even non‐invasive optical stimulation of excitable cells such as neurons, retinal cells, and cardiomyocytes, as demonstrated in recent in vitro and in vivo studies.^[^ ^39^ ^]^ Bioconjugation was confirmed by UV–vis spectroscopy, providing an accurate quantification of the TD molecules conjugated to the phage (363 ± 50, TD molecules/phage) (Table 1) (**Figure** **5**A). The tropism of M13_GD2_TD toward GD2‐positive cells was verified by IF, as described before. Results indicated that M13_GD2_TD maintained specificity for GD2‐positive cells (LAN‐5, CHP‐134, IMR‐32, Kelly and SH‐SY5Y), displaying minimal or absent binding to GD2‐negative lines (SK‐N‐AS, SK‐N‐SH and SK‐N‐BE(2)C) (Figure 5B). Next, the photodynamic activity of M13_GD2_TD was tested on two GD2‐positive cell lines (IMR‐32 and Kelly), and on SK‐N‐AS and SK‐N‐SH used as negative controls. Cells were incubated with various concentrations of M13_GD2_TD (from 0.025 to 1.6 nm phage vector, corresponding to 4.7–300 nm TD equivalents, respectively). After washing, cells were irradiated for 10 min with the previously used white LED source, while negative controls were maintained in the dark. Upon irradiation, ROS generation increased significantly in GD2‐positive cells (IMR‐32 and Kelly), while variations were minimal (SK‐N‐AS) or negligible SK‐N‐SH for GD2‐negative cells, and in the absence of irradiation (Figure 5C). Accordingly, an increase in intracellular ROS was observed in GD2‐positive cells but not in GD2‐negative cell lines (Figure S5A, Supporting Information). The increase in ROS led to lethal photosensitization only for GD2‐positive cells (IMR‐32 and Kelly) in a dose‐dependent manner, while no significant killing was observed in the absence of irradiation; in the case of the GD2‐low‐expressing SK‐N‐AS line, photosensitization mediated by M13_GD2_TD proved weaker, whereas no effect was observed in GD2‐negative SK‐N‐SH cells (Figure 5D). Altogether, these findings demonstrate that M13_GD2_ maintains excellent targeting specificity and photodynamic efficacy after functionalization with different sensitizers, representing a convenient platform for the development of novel NB therapies.

Taking advantage of the intrinsic fluorescence of TD (Figure S5B, Supporting Information), enabling stain‐free tracking of the targeted cells, the cellular events associated with M13_GD2_TD‐mediated photokilling were further investigated. After incubation of the phage vector with IMR‐32 cells and subsequent washing, real‐time confocal microscopy images were acquired every 180 s, using the confocal microscope laser as an irradiation source (an excitation wavelength of 561 nm and an emission wavelength of 550–650 nm). The appearance of membrane blebbing and cell swelling was repeatedly observed upon irradiation (Figure 5E), suggesting the rapid onset of necrotic events consistent with results obtained by the RB‐rigged phage vector (Figure S3B, Supporting Information).

### 2.6 Neuroblastoma Spheroid Penetration and Killing

Multicellular spheroids derived from cancer cells are a preferable option compared to monolayer cell cultures, as they better mimic in vivo tumor characteristics. Poor tissue penetration of drugs, immunotherapeutics, and nanoparticle carriers continues to be a key barrier to solid tumor therapies.^[^ ^40^ ^]^

**Figure 6:**
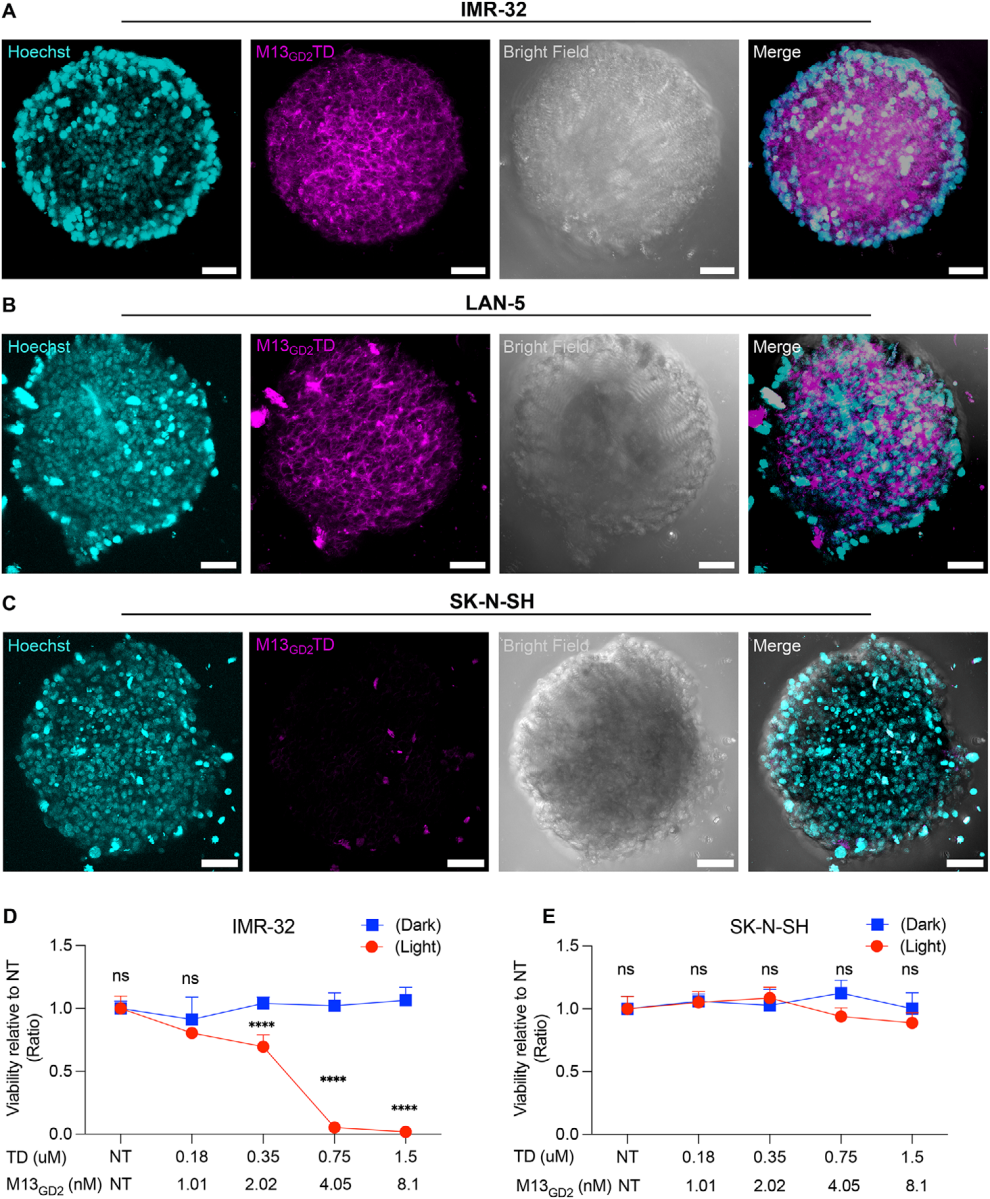
M13_GD2_TD penetration into 3D spheroid models and cytotoxicity. Representation of M13_GD2_TD penetration in **A)** IMR‐32, **B)** LAN‐5, and **C)** SK‐N‐SH generated spheroids. Confocal Z‐stack images were processed and displayed as maximum‐intensity projections. The M13_GD2_TD was visualized in Texas Red using an excitation wavelength of 561 nm and an emission wavelength of 550–650 nm: M13_GD2_TD (magenta) nuclei (cyan). All images were acquired with 20X magnification usingNIKON Eclipse Ti2/A1R confocal microscope. All scale bars represent 50 µm. **D)** CellTiter‐Glo 3D cell viability assay of GD2‐positive IMR‐32‐based spheroids and **E)** GD2‐negative SK‐N‐SH‐based spheroids irradiated with white LED light (red line) (24 mW cm^−^^2^ irradiance) or kept in the dark (blue line). Statistical significance was calculated by two‐way ANOVA followed by Šídák's multiple comparisons test. The significance of differences between light and dark conditions is indicated as ns (not significant, *p* > 0.05), ^*^(*p* < 0.05), ^**^(*p* < 0.01), ^***^(*p* < 0.001), ****(*p* <0.0001).

To expand the therapeutic application of the M13_GD2,_ and investigation of its tumor penetrating ability, NB‐derived 3D models (spheroids) were employed. Due to the higher photostability and fluorescence quantum yield of the photosensitizer, oligothiophene‐based phage bioconjugates were preferred over RB‐bioconjugates (Figure 5E; Figure S5B, Supporting Information). The intrinsic fluorescence of photosensitizers allows the real‐time monitoring of phages and their internalization in 3D models. M13_GD2_TD was tested on three different spheroid models generated from GD2‐positive (IMR‐32 and LAN‐5) and GD2‐negative lines (SK‐N‐SH). After 3 h of M13_GD2_TD incubation, a strong signal was observed on and within the spheroids of both the IMR‐32 and LAN‐5 lines (**Figure** **6**A,B), whereas the fluorescence signal was barely detectable in the SK‐N‐SH spheroid (Figure 6C), suggesting deep penetration of the phage vector only in GD2‐expressing spheroids. The deep penetration of phages in a multicellular spheroid model might overcome intra‐tumoral diffusion resistance, which is a persistent obstacle that significantly reduces the effectiveness of drug delivery and overall cancer treatment outcomes.^[^ ^41^ ^]^ The effect of the concentration of the bioconjugates on cell viability was assessed by performing the PDT treatment on IMR‐32 and SK‐N‐SH spheroids, incubating various concentrations of M13_GD2_TD (from 1 to 8 nm phage vector corresponding to 0.187–1.5 µm TD equivalents). Upon irradiation with white LED light for 10 min (24 mW cm^−^^2^ irradiance), a dose‐dependent photosensitization of the cells in the IMR‐32 spheroid was observed, whereas minimal dark toxicity was found in the non‐irradiated condition (Figure 6D). Conversely, irradiation of the SK‐N‐SH spheroids showed reduced photosensitization under both dark and light conditions (Figure 6E). Overall, these findings support, for the first time, the proficiency of GD2‐targeted phage vectors for non‐invasive treatment of NB.

### 2.7 Safety, Tumor Specificity, and Selective Photodynamic Oncolysis by M13_GD2_RB in Zebrafish

**Figure 7:**
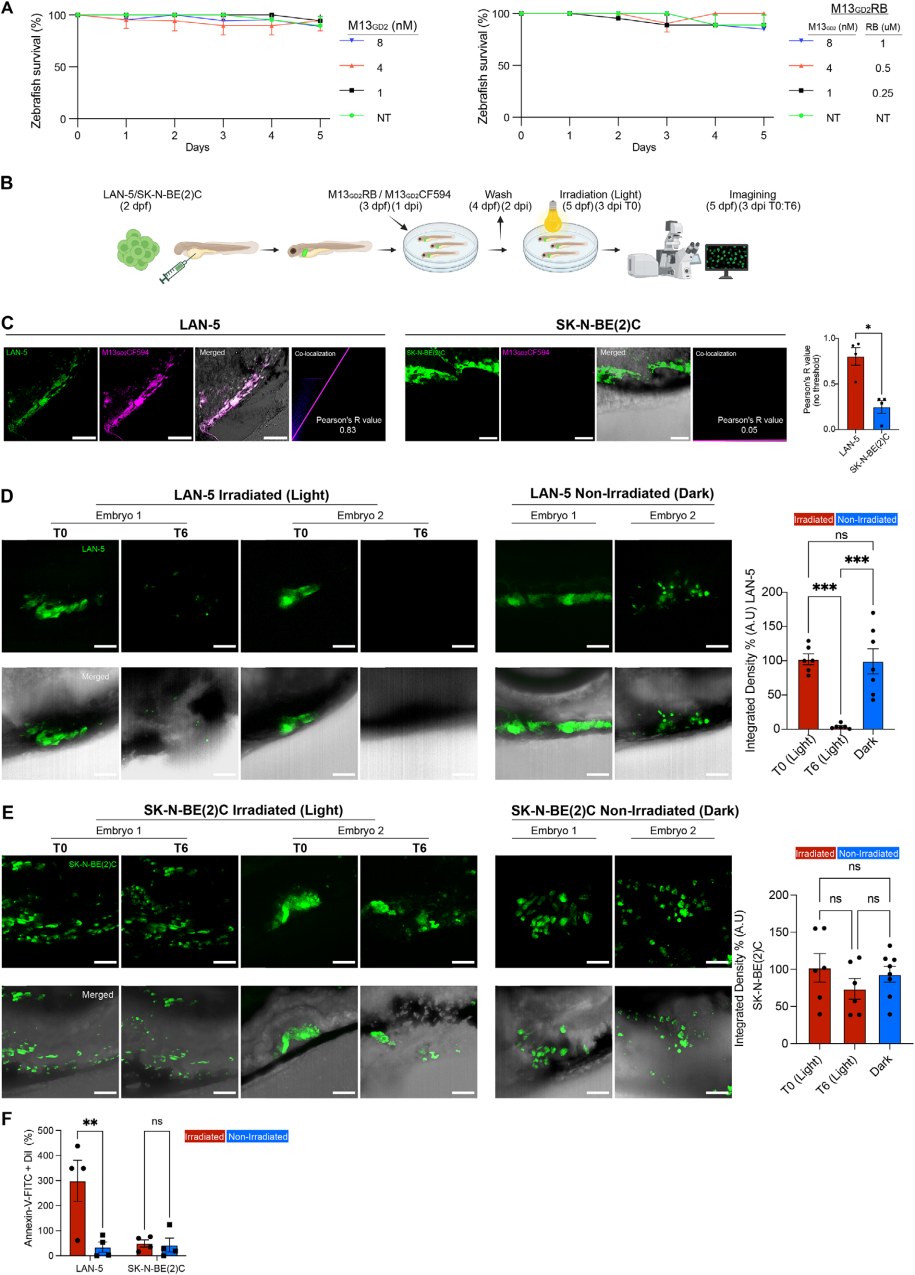
In vivo evaluation of M13_GD2_ and M13_GD2_RB safety, targeting, and photodynamic therapy in zebrafish models. A) To assess the safety of M13_GD2_ and M13_GD2_RB, zebrafish embryos were exposed to different concentrations of M13_GD2_ (1–8 nm) or M13_GD2_RB (1–8 nm phage particles, equivalent to 0.125–1.0 µm RB). An untreated (NT) control group was included. Embryo survival was monitored daily for 5 days. The data represent three independent experiments (*n* = 18). Results are shown as mean ± SEM. **B)** Schematic representation of M13_GD2_CF594‐mediated targeting and photodynamic therapy (PDT) with M13_GD2_RB in zebrafish embryos transplanted with LAN‐5 or SK‐N‐BE(2)C NB cells. **C)** Confocal images showing targeted binding of M13_GD2_CF594 (magenta) to LAN‐5 and SK‐N‐BE(2)C cells (green) in zebrafish embryos. M13_GD2_CF594 was excited at 561 nm and detected at ≈615–640 nm (Texas Red); Vybrant DiO was excited at 488 nm and detected at 500–550 nm. All scale bars: 50 µm. Images were acquired at 20X magnification using aNIKON Eclipse Ti2/A1R confocal microscope. Colocalization was observed within the ROI, as indicated by the magenta line. The *R*‐value represents Pearson's correlation coefficient, calculated using Fiji Coloc2. The data represent two independent experiments (*n* = 4 for each condition). Data are shown as mean ± SEM and were analyzed using an unpaired t‐test. **D)** PDT of LAN‐5 cells in zebrafish. Confocal Z‐stack images at time 0 and 6 h post‐irradiation (Light) show a significant reduction in intensity within the same embryo. Non‐irradiated (Dark) embryos were imaged only once, since RB is photoactivatable. Experiments were repeated twice (*n* = 6). Data are shown as mean ± SEM and were analyzed using one‐way ANOVA. **E)** PDT of SK‐N‐BE(2)C cells in zebrafish. Z‐stack images at time 0 and 6 h post‐irradiation (Light) show no significant reduction in intensity. Non‐irradiated controls were maintained in the dark. Vybrant^TM^ DiO was excited at 488 nm and detected at 500–550 nm. Scale bars: 50 µm. Images were acquired at 20X magnification using a Olympus IX83 P2ZF with Yokogawa CSU‐W1 spinning disk microscope. Data represent two independent experiments (*n* = 6). Data are shown as mean ± SEM and were analyzed using one‐way ANOVA. **F)** Confirmation of photodynamic cell death in LAN‐5 and SK‐N‐BE(2)C cells (Vybrant^TM^ Dil) in zebrafish. Embryos were treated with M13_GD2_RB (1 µm RB equivalent) overnight, washed, and irradiated for 10 min (Light) (24 mW cm^−^^2^ irradiance); controls were kept in the dark. Embryos were dissociated 6 h post‐irradiation and stained with Annexin‐V‐FITC. A total of 50 000 cells per condition were analyzed by flow cytometry. A significant increase in Annexin‐V‐FITC+/Dil+ cells was observed in the irradiated group versus the dark control in LAN‐5 cells, whereas no significant difference was observed in SK‐N‐BE(2)C cells. The data represent four independent experiments (*n* = 40). Data are presented as mean ± SEM and analyzed using two‐way ANOVA followed by Šídák's multiple comparisons test. All significance levels are indicated as ns (not significant, *p* > 0.05), ^*^(*p* < 0.05), ^**^(*p* < 0.01), ^***^(*p* < 0.001), ^****^(*p* < 0.0001).

To determine whether the photolytic phage was also specifically active in vivo, we employed zebrafish embryos as an in vivo model. More specifically, we have used the M13_GD2_RB phage as RB is already FDA‐approved as a therapeutic molecule. First, we evaluated the biocompatibility of the M13_GD2_ in zebrafish embryos. Specifically, 2‐day‐old embryos were exposed to different concentrations of M13_GD2_ (1–8 nm) or M13_GD2_RB conjugates (1–8 nm phage, equivalent to 0.125–1.0 µm Rose Bengal). Importantly, based also on previous observations, phages were conveniently provided to embryos via bath immersion. Embryo survival was monitored daily for 5 days. Across all tested concentrations, survival rates remained comparable and not significantly different from the untreated (NT) control group. These results indicate that both M13_GD2_ and M13_GD2_RB are well tolerated in vivo within the tested dosage range, supporting their safety for further functional downstream applications (**Figure** **7**A **)**. To assess the tumor‐targeting specificity of M13_GD2_ in vivo, the phage was fluorescently labeled with CF594. Zebrafish embryos were xenografted with Vybrant DiO Green‐labeled LAN‐5 (GD2‐positive) or SK‐N‐BE(2)C (GD2‐negative) NB cells at day 2 post‐fertilization (dpf) and subsequently exposed to fluorescently labeled M13_GD2_CF594 (CF594: 2 µM) as outlined in the experimental procedure (Figure 7B). Confocal imaging revealed a strong colocalization between M13_GD2_CF594 (magenta) and LAN‐5 cells (green) (Figure 7C). In contrast, SK‐N‐BE(2)C xenografts showed negligible phage‐associated fluorescence, consistent with their GD2‐negative phenotype (Figure 7C), indicating selective targeting of GD2‐positive NB cells. These qualitative observations were corroborated by quantitative colocalization analysis using Pearson's correlation coefficient, which revealed a significantly higher correlation in LAN‐5 xenografts (*R*‐value 0.83) relative to SK‐N‐BE(2)C (*R*‐value 0.05) (Figure 7C). These findings demonstrate the selective in vivo binding of M13_GD2_ to GD2‐positive NB cells. To explore the therapeutic potential of M13_GD2_, we used RB‐conjugated phages in vivo since RB is FDA‐approved for diagnosing ocular damage and liver function. Its tumor‐photoablation potential in preclinical studies supports translational adaptation for PDT, proving its clinical safety.^[^ ^31^, ^32^, ^33^ ^]^ Zebrafish embryos were xenografted with LAN‐5 or SK‐N‐BE(2)C NB cell lines. As shown in Figure 7B, embryos were microinjected with tumor cells and subsequently treated with 1 µm RB‐conjugated phage. Following overnight incubation of phage bioconjugates in fish water, embryos were washed thoroughly to remove unbound phages and were then maintained in fresh fish water overnight. The subsequent day, embryos were subjected to 10 min light irradiation (24 mW cm^−^^2^ irradiance), followed by Z‐stack confocal imaging at 0 and 6 h post‐irradiation. Strikingly, M13_GD2_RB‐treated LAN‐5 xenografts showed an almost complete loss of Vybrant^TM^ DiO (Green fluorescence) between 0 and 6 h post‐irradiation, as clearly visualized in the same embryo at T0 and T6 (Light); in contrast, non‐irradiated controls (Dark) retained fluorescence comparable to T0 (Light) (Figure 7D **),** indicating effective photoablation. Quantitative analysis revealed a highly significant reduction in integrated intensity between T0 and T6 hours post‐irradiation (Figure 7D). Interestingly, when SK‐N‐BE(2)C xenografts were treated with M13_GD2_RB, no notable reduction in fluorescence was observed following irradiation (Light) (T0 and T6). A minor decrease was observed at T6, which could be due to a slight bleaching of Green fluorescence. However, statistical comparison between time T0 and T6 hours post‐irradiation in both light and dark conditions confirmed no significant difference (Figure 7E **),** suggesting a selective phototoxic effect of M13_GD2_RB toward GD2‐positive cells (LAN‐5). To further validate the selective cytotoxic efficacy of M13_GD2_RB‐mediated PDT, we employed an Annexin V‐FITC binding assay, a canonical marker of cell death. As shown in Figure 7B, zebrafish embryos xenografted with Vybrant DiI‐labeled (red fluorescence) LAN‐5 and SK‐N‐BE(2)C NB cell lines were exposed to M13_GD2_RB treatment. Following photoactivation for 10 min, embryos were enzymatically dissociated into single‐cell suspensions, subjected to serial washing, and stained with Annexin V‐FITC before flow cytometric acquisition. Gating was applied to identify double‐positive cells for DiI (xenografted NB cells) and Annexin V‐FITC. A statistically significant increase in Annexin V and DiI double‐positive signals was observed in LAN‐5 xenografts under light conditions compared to dark controls (Figure 7F **),** confirming that M13_GD2_RB‐mediated photodynamic activation induces cell death, consistent with our previous in vitro demonstration (Figure S3B, Supporting Information). Conversely, SK‐N‐BE(2)C xenografts demonstrated negligible differential Annexin V‐FITC positivity between irradiated and non‐irradiated conditions (Figure 7F **),** as also previously described in (Figure S3B, Supporting Information).

Collectively, these results confirm a light‐dependent, in vivo cytotoxic mechanism that selectively targets GD2‐expressing tumor cells.

## 3 Discussion

In this study, we demonstrate that it is possible to effectively and precisely target GD2‐positive NB cells using an engineered M13 phage. These phage particles serve as high‐payload nanovectors for carrying light‐triggerable drugs that can selectively ablate the GD2‐positive Neuroblastoma cells. The selective killing of GD2‐positive cells by M13_GD2_RB bioconjugates observed in both mixed cell populations of GD2‐positive and GD2‐negative (in vitro) and in zebrafish xenografts (in vivo), addresses the critical challenge of therapeutic specificity and overcomes the limitations associated with the non‐specific targeting observed with current therapies.^[^ ^8a^ ^]^ This approach not only minimizes off‐target effects but also enhances the therapeutic index by maximizing the therapeutic outcome on GD2‐expressing tumor cells.

GD2 disialoganglioside is a well‐established surface antigen and a valuable target for precision medicine in NB,^[^ ^22^ ^]^ particularly given the severe side effects and limitations associated with conventional chemo/radiotherapy.^[^ ^42^ ^]^ The introduction of anti‐GD2 monoclonal antibodies (e.g., dinutuximab) has significantly improved outcomes in high‐risk NB patients, demonstrating prolonged survival both as monotherapy and in combination with chemotherapy.^[^ ^43^ ^]^ In parallel, CAR‐T cells have also been generated to precisely target GD2‐positive NB tumors and have shown some efficacy in relapsed patients.^[^ ^44^ ^]^ Despite their undeniable positive impact on NB treatments, these approaches present several limitations. For instance, the anti‐GD2 antibody is often administered along with IL‐2 and GM‐CSF to enhance its efficacy.^[^ ^45^ ^]^ However, in phase 1 trials, this cocktail resulted in the development of neurotoxicity in adult patients together with hyperleukocytosis, and the production of human antibodies that bind and neutralize the anti‐GD2 antibody, eventually causing hypersensitivity reactions.^[^ ^22^, ^46^ ^]^ Taken together, these findings highlight the importance of GD2 as a promising molecular target for NB therapy but also show that current methods to target GD2‐positive cells require improvement. Compared to purely synthetic scaffolds, engineered biotherapeutics have shown better potential in precision oncology by exploiting multiple tumor‐targeting properties with optimized ablation strategies, enabling improved pharmacodynamics, biocompatibility, and body clearance of the vectors.^[^ ^47^ ^]^ M13 phages offer, by contrast, interesting assets in this scenario: clinically valuable tumor‐targeting moieties (scFv, sdAb, VHH, nanobodies, Darpins, Affibodies, Pronectins, etc) are recurrently selected by phage‐display techniques,^[^ ^48^ ^]^ providing tangible proof of the intrinsic robustness of M13 as a tumor‐targeting vector. In contrast to oncolytic viruses, M13 phages are unable to replicate in eukaryotic cells, with excellent biosafety level 1 (BSL1). Like other phages, we have shown that M13 is highly penetrative in cancer spheroids and can cross biological barriers.^[^ ^26^, ^49^ ^]^ In this respect, it is important to recall that in the zebrafish NB xenograft model, M13_GD2_ phage vectors were added to the fish water, and not injected into the animal or into the NB xenograft/yolk sac (see Materials and Methods). The observation of specific targeting and photo‐oncolysis of GD2‐positive cells only thus provides compelling evidence that the engineered M13 phages can cross biological barriers, without losing their functionalization. In addition to that, our results show that the phage is effective in targeting and penetrating 3D cell cultures. M13_GD2_TD can internalize and efficiently kill GD2‐positive spheroids, whereas no significant killing has been observed in GD2‐negative spheroids. Strikingly, the equivalents of TD loaded onto the phage that yielded effective killing activity were mostly in the nanomolar concentration range.

Our results demonstrate that M13 can be employed to target Neuroblastoma using a nanoarchitectonic design and display of an scFv, derived from FDA‐approved anti‐GD2 antibody.^[^ ^50^ ^]^ By fusing the anti‐GD2 scFv to the pIII minor coat protein (5 copies)^[^ ^51^ ^]^ of the M13 phage, we achieved pentavalent display of the targeting moiety, supporting cooperative binding of the phage to cells, unlike the monovalent scFv. Some studies have reported the lack of efficacy of the anti‐GD2 antibody lies in its limited ability to bind firmly to the GD2 antigen.^[^ ^52^ ^]^

Furthermore, the M13 vector platform has advantages due to its highly tunable surface that can display peptides, fusion proteins, or bioconjugates.^[^ ^25a,b,d^ ^]^ The virion capsid consists of more than 2500 copies of the pVIII protein, each displaying two amines that can be used to covalently bind distinct molecules.^[^ ^53^ ^]^ This feature renders the entire phage a high‐payload carrier with hundreds of conjugation sites for PS, imaging tags, or other molecules.^[^ ^36^, ^53^ ^]^ This is particularly advantageous for PDT applications, where the targeted delivery of sufficient PSs to tumor cells is crucial for maximizing therapeutic efficacy while minimizing off‐target effects. Importantly, the M13_GD2_ nanoarchitecture serves as a proof of principle for loading a variety of therapeutics, whether as single agents or in combination. This opens the possibility of designing a single nanovector capable of delivering hundreds of drug molecules specifically to a single tumor cell, potentially mitigating systemic toxicity typically associated with chemotherapy cocktails. This positions M13 phages as a promising platform within the growing field of targeted biotherapeutics for difficult‐to‐treat cancers.

One limitation of PDT is its restricted tissue penetration, typically up to ≈3 cm, depending on the light wavelength. However, this issue can be addressed with the use of thin optical fibers, as exemplified by Tookad, a recently approved PDT modality for the treatment of low‐risk prostate cancer, which employs thin optical fibers connected to a laser, reaching the deeper cancerous area with the help of magnetic resonance imaging and ultrasound guidance.^[^ ^54^ ^]^ The approach has been successfully applied in several clinical contexts, including pancreatic and head‐and‐neck cancers.^[^ ^55^ ^]^ These devices can deliver higher irradiance than standard LEDs, allowing efficient photosensitizer activation within very short exposure times, even under in vivo conditions,^[^ ^56^ ^]^ thus minimizing treatment duration and reducing potential off‐target effects. An alternative strategy to overcome the light‐penetration issue against deeper‐seated tumors is offered by the functionalization with PSs responsive to near‐infrared (NIR) light. NIR light, particularly in the 650–900 nm (NIR‐I) window, exhibits superior tissue penetration and is increasingly used in clinical phototherapies. By substituting our current photosensitizers with NIR‐responsive analogues, we might retain the phage's targeting and delivery advantages while overcoming the depth limitations of visible light.

Another key clinical challenge is the fact that not all NBs express GD2 and that several GD2‐positive NBs treated with the anti‐GD2 monoclonal antibody relapse and progress toward more aggressive phenotypes, in part because of the loss of GD2 surface expression.^[^ ^52^, ^57^ ^]^

Our study addressed this critical issue by first finding that among the genes required to synthesize GD2 and its intermediates, *ST8SIA1* is consistently silenced in GD2‐negative NB cell lines. Second, we designed a molecular genetic strategy to reactivate *ST8SIA1* expression in a GD2‐negative NB cell line through selective repositioning of a dCAS9‐VPR‐gRNA complex near the transcription start site of the silenced gene. This targeted gene reactivation approach effectively converted resistant GD2‐negative cells into GD2‐expressing ones. Furthermore, *ST8SIA*1 re‐expression and restoration of GD2 were sufficient to make those cells sensitive to M13_GD2_RB PDT. These findings suggest that re‐inducing GD2 expression in relapsed tumors could offer a powerful approach to restore sensitivity to GD2‐targeted therapies.

Finally, GD2‐targeting phages may be employed beyond NB. GD2 is ranked the 12th most important cancer antigen, due to its immunogenicity, therapeutic potential, and expression profile. It is also found in other malignancies including melanoma,^[^ ^58^ ^]^ glioma,^[^ ^59^ ^]^ retinoblastoma,^[^ ^60^ ^]^ medulloblastoma,^[^ ^61^ ^]^ small‐cell lung cancer,^[^ ^62^ ^]^ breast cancer,^[^ ^63^ ^]^ sarcoma,^[^ ^64^ ^]^ bladder cancer,^[^ ^65^ ^]^ colorectal cancer,^[^ ^66^ ^]^ and prostate cancer.^[^ ^67^ ^]^

In conclusion, our results propose a novel methodology to specifically target GD2‐positive tumors with high killing selectivity, potentially with fewer side effects compared to other more conventional therapies. Together, these innovations suggest that engineered M13 phages could become valuable components in the next generation of precision oncology treatments.

## 4 Experimental Section

### Cell Culture

The following human cell lines were used in this study: SK‐N‐BE(2)C, IMR‐32, SH‐SY5Y, SK‐N‐SH, SK‐N‐AS, HEK293t, CHP‐134, Kelly, and LAN‐5 were a gift from Prof. Michelle Haber (Children's Cancer Institute Australia). LAN‐5, CHP‐134, Kelly and SK‐N‐SH were cultured and maintained in RPMI 1640 supplemented with heat‐inactivated fetal bovine serum 10% (FBS), 1% L‐Glutamine (200 mm), and 1% penicillin‐streptomycin (100 U/mL), while IMR‐32, SH‐SY5Y, SK‐N‐AS, SK‐N‐BE(2)C, and HEK293t, were cultured in high‐glucose Dulbecco's Modified Eagle Medium (DMEM), 10% FBS, and 1% penicillin–streptomycin (100 U mL^−1^). All cell lines were grown in a humidified incubator containing 5% CO_2_ at 37 °C.

### Immunofluorescence of GD2

Glass coverslips were placed in a 6‐well plate (1 × 10^6^ cells per well) and coated with collagen‐containing 0.02% acetic acid and left for 1 h at room temperature. The coverslips were gently rinsed twice with phosphate‐buffered saline (PBS) before seeding cells (LAN‐5, CHP‐134, IMR‐32, Kelly, SH‐SY5Y, SK‐N‐AS, SK‐N‐SH, SK‐N‐BE(2)C) and allowed to proliferate overnight until reaching 80% confluency. The next day, the culture media were removed, and cells were washed with PBS 1X. Subsequently, the cells were fixed with 4% Paraformaldehyde (PFA) in PBS 1X at room temperature for 15 min. Afterward, cells were briefly washed with PBS containing 0.05% Tween 20 and blocked with 4% normal donkey serum (NDS) for 45 min at room temperature. The primary antibody anti‐GD2 14G2a (1:300, sc‐53831, Santa Cruz) was incubated overnight at 4 °C. The next day, cells were treated with a FITC‐conjugated anti‐mouse secondary antibody for 1 h at room temperature, and nuclear staining was carried out with Hoechst 33342(1 µg mL^−1^) for 10 min, and coverslips were mounted in antifading media. IF experiments were checked with a fluorescence conventional microscope (Nikon Eclipse 90i), and representative images were acquired using a 40X magnification at a NIKON Ti2/A1R confocal microscope using laser excitation at 488 nm (FITC) and 405 nm (Hoechst), and Fiji software was used for analysis.

### Flow Cytometry Analysis of GD2

The quantitative expression analysis of GD2 was carried out by flow cytometry. A total of 1 × 10^6^ cells of each cell line (LAN‐5, CHP‐134, IMR‐32, Kelly, SH‐SY5Y, SK‐N‐AS, SK‐N‐SH, SK‐N‐BE(2)C) were harvested and resuspended in cold PBS 1X. Cells were fixed with 4% PFA, resuspended in PBS 1X for 15 min at room temperature, followed by three washes with PBS 1X + Tween 20 0.05% (Washing Buffer, WB). Subsequently, all cell lines were incubated for 45 min with anti‐GD2 mAb (1:300, sc‐53831, Santa Cruz Biotechnology) to label GD2, while normal mouse IgG (1:300, sc‐2025, Santa Cruz) was used as a negative control for GD2 expression. Subsequently, cells were washed three times by centrifugation with WB. After that, cells were incubated with FITC‐conjugated secondary anti‐mouse IgG (Jackson ImmunoResearch) for 45 min in the dark and subsequently washed with WB. All phases of the experiments were carried out at 4 °C. The samples were immediately analyzed with a Bio‐Rad flow cytometer. For each cell line, a minimum of 10000 events were collected, and analysis was performed by FlowJo software.

### Cloning of Phagemid and M13_GD2_ Production

The single‐chain fragment variable (scFv) sequences that recognized the GD2 were obtained from 14G2a human‐mouse chimeric antibody, and the VH and VL were linked by (Gly_4_Ser)_3_ linker. The gene encoding the anti‐GD2 scFv was synthesized by OfficinaeBio (part ID: 34O6QQZ) and amplified by PCR using oligonucleotides AD315 (GTTTTTGAGCTCGAAGTTCAGCTGC) and AD316 (GTTTTTACTAGTTTTCAGTTCCAGTTTAGTACC). The resulting amplicons were purified and digested with SacI and SpeI (New England Biolabs). Next, the DNA segment was cloned into the SacI/SpeI‐linearized pPK3DsbA plasmid, in which the ompA leader sequence found in the original pComb3XSS vector, a gift from Carlos Barbas (Addgene plasmid # 63890) was replaced with a dsbA leader sequence, while the SacI‐SpeI insert encompassing the original light and heavy chain stuffers was replaced by a multiple cloning site (MCS), in frame with the C‐terminal domain of the pIII protein^[^ ^25a,b^ ^]^ generating pCOMB3xss‐scFv anti‐GD2 phagemid. Positive clones were validated by Sanger sequencing of the phagemid. M13_GD2_ phage was produced by co‐transformation of *E. coli* DH5α with pCOMB3xss‐scFv anti‐GD2 phagemid and helper phage plasmid derivative M13KO7Δg3pΔoriF1 (GeneBank OQ803484.1), in *E. coli* DH5α. Co‐transformants were grown in LB supplemented with Ampicillin (100 µg mL^−1^), kanamycin (25 µg mL^−1^), and 0.4 mm isopropyl ß‐D‐1‐thiogalactopyranoside (IPTG) for 24 h at 32 °C. Phage purification was performed as previously described.^[^ ^25a^ ^]^

Briefly, the bacterial culture was centrifuged at 10 000g for 20 min at 4 °C to remove bacteria. The supernatant was centrifuged again for 5 min to pellet the remaining bacteria, and the supernatant containing M13_GD2_ was gently transferred to fresh bottles. Next, the supernatant was supplemented with 4% (w/v) of polyethylene glycol (PEG) 8000 and 3% (w/v) NaCl and incubated for 90 min at 4 °C. The supernatant containing M13_GD2_ /PEG 8000/ NaCl was again centrifuged at 10 000g for 20 min at 4 °C. Pelleted phages were gently resuspended in PBS 1X, followed by lowering the pH to 4.2 with 0.5 m HCl to reach the phage isoelectric point (IEP). Next, the solution was centrifuged at 14,000g for 15 min at 4 °C, followed by gentle resuspension of the pellet in sterile PBS 1X. The additional IEPs were performed to eliminate the endotoxins derived from the bacterial culture for downstream applications. Phage concentrations were calculated by measuring the absorbance at 269 nm in a UV–vis spectrophotometer (Agilent Cary‐60, Agilent, Santa Clara, CA, USA) using an extinction coefficient of *ε* = (3.84 cm^2^ mg^−1^).

### scFv Anti‐GD2 Nucleotide Sequence

5′GAAGTTCAGCTGCTGCAATCTGGTCCAGAACTGGAGAAACCGGGTGCGTCTGTTATGATCTCTTGTAAAGCATCTGGCTCCAGCTTCACCGGTTACAACATGAACTGGGTTCGTCAGAACATCGGCAAAAGCCTGGAATGGATCGGCGCGATCGACCCATACTATGGCGGCACCTCTTACAACCAGAAATTCAAAGGTCGCGCTACGCTGACTGTCGATAAATCTTCTTCCACCGCATATATGCACCTGAAAAGCCTGACTTCTGAAGACTCTGCCGTATACTACTGCGTTAGCGGTATGGAATACTGGGGTCAGGGTACTTCCGTTACCGTTTCTGGTGGTGGCGGTTCTGGTGGTGGTGGTTCTGGTGGTGGCGGTAGCGATGTTGTGATGACCCAGACCCCTCTGAGCCTGCCGGTATCCCTGGGTGACCAGGCTTCTATCTCTTGCCGTTCCTCTCAGTCTCTGGTTCATCGCAATGGTAACACCTATCTGCACTGGTATCTGCAGAAGCCGGGTCAGTCCCCAAAACTGCTGATTCATAAAGTTTCTAACCGTTTCTCTGGTGTTCCGGACCGTTTCTCCGGTTCTGGCAGCGGTACTGATTTCACCCTGAAAATTAGCCGCGTCGAAGCTGAAGACCTGGGCGTGTACTTTTGTTCTCAGTCCACCCACGTGCCGCCACTGACCTTTGGTGCTGGTACCAAACTGGAACTGAAA 3′

### Western Blotting

The confirmation of scFv anti‐GD2 fused with pIII was validated by immunoblotting. M13_GD2_ phages (2 × 10^10^) were electrophoresed in a 12% sodium dodecyl sulfate‐polyacrylamide gel electrophoresis (SDS‐PAGE). Proteins were then transferred to the nitrocellulose membrane for 2 h at 260 mA at 4 °C. Membranes were blocked for 1 h at room temperature in blocking buffer (PBS 1X, pH 7.4, 5% milk, 0.05% Tween). Primary antibodies, anti‐GD3 synthase antibody (B‐11, sc‐390123; Santa Cruz Biotechnology, 1:100), and anti‐M13 pIII (New England BioLabs, Ipswich, MA, USA, 1:400) were incubated overnight at 4 °C. Post‐incubation, the membrane was washed three times with washing buffer (PBS 1X pH 7.4, 2.5% milk, and 0.05% Tween). The membrane was then incubated with the horseradish peroxidase HRP‐conjugated anti‐mouse IgG secondary antibody (Jackson ImmunoResearch Laboratories, West Grove, PA, USA) (1:800) for 1 h at room temperature. The membrane was developed using enhanced chemiluminescence (ECL) solution (1.25 mm luminol in Tris 100 mm pH 8.8; 6.8 mm coumaric acid; 30% H_2_O_2_). Images were acquired by ChemiDoc (Bio‐Rad).

### Characterization of M13_GD2_ and M13 Wild Type by AFM

AFM was performed on a Multimode‐8 microscope (Bruker, USA). Hydrated phages (10^13^/mL) diluted in PBS were adsorbed for 2 min on freshly cleaved mica (Electron Microscopy Sciences). Imaging in liquid was carried out with ScanAsyst Fluid+ probes in Peak‐Force Tapping mode. Micrographs were flattened with Nanoscope Analysis (v 1.80). (x, y, z) coordinates of hundreds of individual phages were digitized semi‐automatically with a custom MATLAB script as described in.^[^ ^68^ ^]^

### Optimization of Concentration and Validation of Refactored M13_GD2_ With Confocal Microscopy

Before binding assays, optimal M13_GD2_ concentrations were determined on GD2‐positive cells (IMR‐32 and Kelly) and GD2‐negative cells (SK‐N‐SH). Various concentrations of M13_GD2_ (nm: 1.66, 0.166, 0.016) were tested. The maximal binding was demonstrated by 1.66 nm of M13_GD2_ and was used thereafter. M13 wild‐type (M13_WT_) was used as a control. Cells (1 × 10⁶ per well) were incubated with 1.66 nm M13_GD2_ or M13_WT_ for 45 min at room temperature, washed twice with washing buffer, fixed in 4% PFA for 15 min, blocked, and stained with anti‐M13/fd/F1 (1: 300, Progen) overnight at 4 °C, followed by Cy3‐labeled anti‐mouse IgG (1: 600) for 1 h. Nuclei were counterstained with Hoechst 33342 (1 µg mL^−1^). Images were acquired on a NIKON Ti2/A1R confocal microscope (40X) and analyzed with Fiji.

### Flow Cytometry Analysis of M13_GD2_ and M13_WT_

For flow‐cytometric validation, 1 × 10⁶ cells of each line were fixed with 4% PFA for 15 min and washed. Subsequently, cells were incubated with mouse monoclonal anti‐M13/fd/F1 filamentous phage (Progen) (dilution 1:300) for 45 min at 4 °C and then with FITC‐conjugated anti‐mouse IgG (Jackson ImmunoResearch) for 45 min in dark conditions, followed by washing with WB. The samples were immediately analyzed by using a Guava EasyCyte flow cytometer. For each cell line, a minimum of 10 000 events were collected, and analysis was performed by FlowJo software.

### Conjugation of CF488A and CF594 to the M13_GD2_ Phages

CF488A‐SE or CF594 was dissolved in anhydrous DMSO to reach a 10 mm concentration. 17 µL of the solution was added dropwise to 1 mL of a 100 mm sodium carbonate buffer (pH 9) solution containing M13_GD2_ phages at a concentration of 40 nm. The reaction proceeded for 3 h at 25 °C under constant shaking at 700 rpm (Thermo‐Mixer HC, S8012‐0000; STARLAB, Hamburg, Germany) in dark conditions. Bioconjugate (M13_GD2_CF488 or M13_GD2_CF594) were purified by dialysis.

### Conjugation of TD and RB to the M13_GD2_ Phages

TD was dissolved in anhydrous DMF to 5 mm, and 50 µL were added dropwise to 1 mL of 40 nm M13_GD2_ in sodium‐carbonate buffer (100 mm, pH 9) with vigorous stirring. After 3 h at 25 °C (700 rpm, dark), samples were centrifuged at 14 000 g for 10 min to remove insoluble TD, dialyzed extensively, and centrifuged again. RB activation and conjugation followed optimized procedures.^[^ ^25a,b^ ^]^

### Purification of the Bioconjugate Phages by Dialysis

To remove the unconjugated dyes and reaction coproducts, the M13_GD2_ bioconjugates were purified via dialysis versus 100 mm sodium carbonate buffer (pH 9), using a regenerated cellulose membrane (14 kDa cut‐off) and kept in dark conditions. UV–vis spectra of the dialysate were performed at each step to monitor the purification process. The last dialysis cycle was performed against PBS 1X to restore the physiological conditions (**Table** **1**).

**Table 1:** Degree of conjugation of RB, TD, CF594, and CF488 with M13_GD2_.

	Phages	Phage [v/mL]	Phage [nM]	Dye [µM]	Ratio [dye/phage]	SD ratio
1	M13_GD2_RB	1.20E + 13	20.6	4.7	228	50
2	M13_GD2_TD	2.05E + 13	34.1	12.4	363	40
3	M13_GD2_CF594	2.15E + 13	36	12.9	360	43
4	M13_GD2_CF488	6.88E + 12	11.4	2.3	202	50

### Validation of M13_GD2_RB and M13_GD2_TD With Confocal Microscopy

Bioconjugate targeting was assessed to check the loss of affinity after functionalization. Cells (5 × 10⁴ per coverslip) were incubated with M13_GD2_RB or M13_GD2_TD for 45 min at room temperature. Subsequently, the cells were washed and fixed with 4% PFA for 15 min and blocked with blocking solution (PBS+0.05%tween20+4%NDS). After blocking, the cells were stained with FITC‐conjugated anti‐M13/fd/F1 (1: 300) (Progen). Nuclei were counterstained with Hoechst 33342 (1 µg mL^−1^). Live‐cell imaging of M13_GD2_TD intrinsic TD fluorescence was performed after 45 min incubation. The coverslips were then washed and fitted into an Attofluor cell chamber (Invitrogen) supplemented with complete DMEM medium without red phenol (cat no: 21063029). TD was excited at 561 nm and emission collected at 550–650 nm on a NIKON Eclipse Ti2/A1R microscope.

### Photodynamic Treatment of 2D Cell Cultures

For PDT, two GD2‐high (IMR‐32, Kelly), one GD2‐low (SK‐N‐AS) and two GD2‐negative (SK‐N‐BE(2)C, SK‐N‐SH) lines were used. Cells (5 × 10⁴ per well) were seeded in 96‐well plates. After 24 h, cells were incubated for 90 min with M13_GD2_RB (50, 25, 12 nM RB: 180, 90, 45 pM phage) or M13_GD2_TD (300–4.6875 nM TD: 1.6–0.025 nM phage). Unbound phage was removed by washing with DMEM medium without red phenol, and cells were irradiated with white LED light (Valex cold white LED, 24 mW cm^−^^2^ irradiance) for 15 min RB‐conjugates or 10 min TD‐conjugates at 30 cm; controls were kept in the dark. Medium was replaced, and cells were incubated for 24 h before assays. Control experiments were conducted under the same conditions I), cells were exposed to light (irradiation) with concentrations of M13_GD2_RB and M13_GD2_TD (Phage toxicity), II) the cells were exposed to light (Phototoxicity) III), the cells were kept in the dark with M13_GD2_RB and M13_GD2_TD without irradiation (Dark phage toxicity), IV) the cells were kept in dark (Dark toxicity).

### Cell Viability Assay (MTT)

Both for the M13_GD2_RB and M13_GD2_TD induced photodynamic cell death, cell viability was determined using the 3‐[4,5‐dimethylthiazole‐2‐yl]‐2,5‐diphenyltetrazolium bromide (MTT) assay after 24 h of post‐PDT. Gently, the medium was removed from the 96‐well plate, and the freshly added medium contained a final concentration of MTT 0.5 mg mL^−1^. Cells were incubated for 90 min in a humidified incubator at 37 °C containing 5% CO_2_. After incubation, the insoluble product formazan produced by the reduction of MTT by NADPH‐dependent oxidoreductases expressed in live cells was solubilized in dimethyl sulfoxide (DMSO) at room temperature and agitated for 10 min in the dark.^[^ ^69^ ^]^ The absorbance was then measured at wavelengths of 570 and 690 nm by the VICTOR3 (1420 Multilabel Counter‐PerkinElmer, Turku, Finland).

### Real‐Time Monitoring of the Photokilling by M13_GD2_TD

Photokilling activity of M13_GD2_TD was real‐time monitored using a confocal microscope. IMR‐32 cells were seeded at a density of 50 000 cells on poly‐l‐lysine (PLL) (1 mg mL^−1^) coated coverslips and incubated overnight to allow for cell adhesion and proliferation. Subsequently, the cells were treated with M13_GD2_TD (2 nM phage vector, corresponding to 2 µm of TD) along with Hoechst 33258 (2 µg mL^−1^) in a complete medium for 90 min in a humidified incubator. Next, unbound M13_GD2_TD was washed with PBS 1X and kept in DMEM without red phenol. Confocal time‐lapse images were acquired every 2.5 min for 30 min with 30 s intermittent irradiation. TD excitation 561 nm, emission 550–650 nm; analysis in Fiji.

### 3D Spheroids and Confocal Imaging

For spheroid formation, IMR‐32 (GD2‐positive), LAN‐5 (GD2‐positive), and SK‐N‐SH (GD2‐negative) cells were seeded in Ultra‐low attachment (ULA) U‐shaped 96‐well plate. In brief, 1000 cells of IMR‐32, LAN‐5 (GD2‐positive), and SK‐N‐SH were seeded per well in DMEM and RPMI complete medium supplemented with 10% FBS, and 1% penicillin–streptomycin (100 U mL^−1^), respectively. The ULA plates were then centrifuged at 400g for 5 min to bring the cells into close contact for rapid spheroid formation. The cells were maintained in the incubator for 5 days and then exposed to a maximum concentration of M13_GD2_TD (4 nm phage vector, corresponding to 0.75 µm of TD) for 3 h along with Hoechst 33258 (2 µg mL^−1^). Next, spheroids were washed with PBS 1X and maintained in DMEM without red phenol. Images were acquired by NIKON Eclipse Ti2/A1R confocal microscope using ND‐acquisition z‐stack and were processed and displayed as maximum intensity projections.

### Photodynamic Treatment and CellTiter‐Glo 3D Cell Viability Assay

Pre‐generated spheroids from IMR‐32 and SK‐N‐SH were incubated with various concentrations of M13_GD2_TD for a maximum of 3 h in a ULA plate. Spheroids were washed, irradiated with white LED light (Valex cold white LED, 24 mW cm^−^^2^ irradiance) from a 30 cm distance for 15 min, and maintained in DMEM without red phenol. After treatment, the medium was replaced with a complete medium. Control experiments were carried out in the same setup. After 24 h of post‐treatment, the spheroid viability was determined using CellTiter‐Glo 3D Cell Viability Assay kit (Promega, Cat. #G9681). In brief, the spheroids were treated with M13_GD2_TD‐mediated PDT, and after 24 h, the spheroids were transferred from a ULA 96‐well plate to a 96‐well opaque culture plate. Gently, the medium was removed, and spheroids were resuspended in 100 µL of CellTiter‐Glo 3D reagent and incubated for 30 min at 37 °C. Next, the plate was shaken for 5 min to ensure the reagent penetrated the spheroids. Finally, the luminescence signal was recorded with VICTOR3 (1420 Multilabel Counter‐PerkinElmer, Turku, Finland), which is proportional to the amount of ATP present, correlating with the number of viable cells.

### Extracellular ROS Detection Using ROS‐Glo H_2_O_2_ luminescence Assay

Extracellular ROS production was determined by following the manufacturer's protocol (Progema, G8820) using the ROS‐Glo H_2_O_2_ kit. GD2‐positive cells (IMR‐32 and Kelly), and GD2‐negative cells (SK‐N‐AS, SK‐N‐SH, SK‐N‐BE(2)C) were seeded in a 96‐well plate. The following day, the cells were treated with different concentrations of M13_GD2_RB (ranging from 50, 25, and 12 nm RB, corresponding to 180, 90, and 45 pm of phage vector equivalents, respectively) or with various concentrations of M13_GD2_TD (ranging from 300, 75, 18, 4.0 nm TD, corresponding to 1.6, 0.4, 0.1, 0.02 nm phage vector, respectively). Subsequently, the cells treated with M13_GD2_RB or M13_GD2_TD were irradiated with white LED light (Valex cold white LED, 24 mW cm^−^^2^ irradiance) for 15 min and 10 min, respectively. Following irradiation, the cells were incubated at 37 °C for 30 min with a 25 µm H_2_O_2_ substrate provided in a dilution buffer. Next, ROS‐Glo detection agent was added (100 µL), consisting of D‐cysteine (1 µL) and signal enhancer solution, and mixed gently by shaking the plate. Plates were then incubated for 20 min at the benchtop to stabilize the luminescent signal. The luminescence signal is proportional to the amount of H_2_O_2_ present in the sample and was recorded by VICTOR3 (1420 Multilabel Counter‐PerkinElmer, Turku, Finland).

### Detection of Intracellular ROS Production

Intracellular ROS accumulation was measured using H_2_DCFDA, which undergoes cleavage by intracellular esterases, transforming into non‐fluorescent H_2_DCF [91]. Briefly, GD2‐positive cells (IMR‐32 and Kelly), and GD2‐negative cells (SK‐N‐AS, SK‐N‐SH, SK‐N‐BE(2)C) were seeded in a 96‐well plate. The following day, the cells were treated with different concentrations of M13_GD2_RB (50 and 25 nm RB, corresponding to 180 and 90 pm of phage vector equivalents, respectively) or with various concentrations of M13_GD2_TD (ranging from 300, 75, 18 nm TD corresponding to 1.6, 0.4, 0.1 nm of phage vector equivalents, respectively). Subsequently, the cells were irradiated with white LED light (Valex cold white LED, 24 mW cm^−^^2^ irradiance) for 15 min M13_GD2_RB and 10 min M13_GD2_TD. Next, 2 µm H_2_DCFDA probes were resuspended in DMEM media (without red phenol) and added to irradiated cells, then the plate was returned to the incubator for a further 30 min of incubation. Next, the cells were fixed in the well with 50% trichloroacetic acid (TCA) dissolved in the growing medium for 1 h at 4 °C, washed 5 times with water, and dried at room temperature. The attached cells were incubated for 30 min with 0.4% SRB diluted in 1% acetic acid at room temperature. Subsequently, the wells were washed 5 times with 1% acetic acid to remove the excess dye. The dye bound to the cells was finally solubilized with 10 mm Tris (pH 10.5), and the relative absorbance of SRB was detected using a plate reader VICTOR3 (1420 Multilabel Counter‐PerkinElmer, Turku, Finland) at the wavelength of 560 nm.

### Annexin‐V Assay 2D Culture

Annexin‐V‐FITC and propidium iodide (PI) double staining were used to determine the cell death mechanism mediated by M13_GD2_RB. Twenty‐four hours post‐PDT, cells were harvested, washed, and resuspended in annexin binding buffer (500 µL). Annexin‐V‐FITC (5 µL) was added for 10 min at 4 °C, followed by PI (1 µg mL^−1^) for 5 min; samples were analyzed by flow cytometry.

### Cloning and Plasmids

A stable cell line of PB‐TRE dCas9‐VPR in SK‐N‐BE(2)C was generated by co‐transfection of the Super PiggyBac Transposase and the PiggyBac Vector (System Biosciences, Palo Alto, CA, USA). This was done using a 1:5 ratio and the transfection reagent Effectene from QIAGEN, following the manufacturer's instructions. Cells expressing the construct were selected and maintained in 400 µg mL^−1^ Hygromycin B until untransfected cells were no longer viable. Lentiviruses carrying the sgRNA were produced in HEK‐293 packaging cells by co‐transfecting the pLenti‐sgRNA plasmid (Addgene plasmid #71409) along with helper plasmids psPAX2 (Addgene plasmid #12260) and pMD2.G (Addgene plasmid #12259). After 72 h, the viral supernatant was collected and used to infect the PB‐TRE dCas9‐VPR SK‐N‐BE(2)C cells with a Multiplicity of Infection (MOI) of 0.5. Polybrene was used as a transduction agent. Cells expressing the construct were selected and maintained in 0.5 µg mL^−1^ Puromycin selection for 5 days. To create the constructs encoding the sgRNA for the ST8SIA1 gene, two complementary oligonucleotides were annealed, phosphorylated, and inserted into the pLenti‐sgRNA plasmid after it was digested with BsmBI. The oligonucleotide sequences were:

‐295 sgRNA ST8SIA1:

Fwd: (5′→3′) TCTGGGCGAGTGTCGCGGCT

Rev: (5′→3′) AGCCGCGACACTCGCCCAGA

‐20 sgRNA ST8SIA1:

Fwd:(5′→3′) CGGCGCAGAGAGCGCGTCTC

Rev: (5′→3′) GAGACGCGCTCTCTGCGCCG

sgRNA scrambled:

Fwd: (5′→3′) GCTTAGTTACGCGTGGACGA

Rev: (5′→3′) TCGTCCACGCGTAACTAAGC

### RT‐qPCR

Total RNA was extracted using TRI Reagent (Sigma–Aldrich). The RNA was then precipitated and resuspended in 50 µL of Molecular biology‐grade water (Sigma–Aldrich). To remove any DNA contamination, 5 µg of RNA were treated using the DNA‐free Removal kit (Invitrogen). Subsequently, 1 µg of RNA was reverse‐transcribed into complementary DNA (cDNA) using the iScript reverse transcription supermix kit (BIO‐RAD). qRT‐PCR was performed using 5 µg of cDNA, and the SSO Advanced Universal SYBR Green supermix from BIO‐RAD.

Primer sequences: ST8SIA1:

Fwd:(5′→3′): GGCTGTGGCCGTCAAATAGA

Rev: (5′→3′): TGCTGGGATTAGCTGTCACT

### Photodynamic Treatment of dCAs9‐VPR‐SK‐N‐BE(2)C Cells With M13_GD2_RB

GD2 expression in dCAs9‐VPR‐SK‐N‐BE(2)C was induced by 1 µg mL^−1^ doxycycline (Dox) for 72 h. Next, 50 000 cells were incubated with various concentrations of M13_GD2_RB (ranging from 50, 25, 12 nm RB, corresponding to 180, 90, 45 pm of phage vector, respectively) for 90 min. Cells were washed three times with DMEM without red phenol to eliminate unbound M13_GD2_RB and irradiated with white LED light (Valex cold white LED, 24 mW cm^−^^2^ irradiance) from a 30 cm distance for 15 min. Immediately after irradiation, the cell medium was replaced, and the cells were returned to the incubator for 24 h. Control experiments were conducted under the same conditions. I) Cells were exposed to various concentrations of M13_GD2_RB with irradiation (phage toxicity), II) Cells were exposed to light (Phototoxicity), III) Cells were kept in the dark with M13_GD2_RB without irradiation (Dark phage toxicity), IV) sgRNA scrambled, −295 sgRNA, and −20 sgRNA (± Dox) in non‐irradiated conditions (Dark toxicity). Survival rates were determined by MTT assay (as described above) post 24 h PDT treatment.

### Co‐Culture and Dual Luciferase Assay

Luciferase reporter activity was measured using the Dual Luciferase Assay System (E1980, Promega, Madison, WI, USA). Chemiluminescence values for Firefly (*Photinus pyralis, Pp*) and Renilla (*Renilla reniformis, Rr*) luciferases were measured with a GloMax 20/20 instrument (Promega). Briefly, Kelly (GD2‐positive, constitutively expressing *Renilla reniformis* luciferase) and SK‐N‐AS (GD2‐negative, constitutively expressing firefly luciferase) cells were co‐cultured, followed by incubation with M13_GD2_RB and PDT. Post‐PDT (24 h), the cells were collected and washed with PBS 1X, and Passive Lysis Buffer 1X (PLB) (E194A) was added to each well, with constant agitation for 20 min. 15 µL of cell lysate was collected in a 1.5 mL tube, and 35 µL of luciferase assay reagent II (LAR II) was added. Subsequently, Firefly luciferase activity was measured. Following this, 35 µL of Stop & Glo was resuspended with cells and Renilla luciferase activity was measured to assess survival.

### Animal Care

All zebrafish experiments were conducted in compliance with European Directive 2010/63/EU and Italian legislation governing animal welfare. Authorization for animal experimentation was granted by the Ethics Committee of the University of Padua and the Italian Ministry of Health (Authorization no. 1111/2024). Wild‐type *Danio rerio* (zebrafish) were housed at a controlled temperature of 28.5 °C under a 12 h light/dark cycle. Feeding protocols followed the guidelines established by.^[^ ^70^ ^]^ For anesthesia and euthanasia of embryos and larvae, tricaine was administered in the water at concentrations of 0.16 and 0.3 mg mL^−1^, respectively. The Tuebingen wild‐type strain was used throughout, and all procedures adhered to standard zebrafish handling protocols.^[^ ^71^ ^]^

### Xenotransplantation

At 2 days post‐fertilization (dpf), embryos were manually dechorionated, anesthetized with 0.16 mg mL^−1^ tricaine, and positioned in plastic grooves filled with 2% methylcellulose in PBS. NB cells were labeled for 20 min at 37 °C using Vybrant^TM^ DiI or Vybrant^TM^ DiO Cell‐Labeling Solution (5 µg mL^−1^, Thermo Fisher Scientific), a lipophilic dye that uniformly stains the cell membrane. Following staining, cells were suspended in 10 µL PBS or the appropriate culture medium at a concentration of 1 × 10^5^ cells µL^−1^, loaded into glass capillary needles, and microinjected into the yolk sac of each embryo (≈200 cells per embryo) using a WPI PicoPump device. Post‐injection, embryos were incubated at 33 °C and monitored daily until 3 days post‐injection (dpi), the experimental endpoint. Dead embryos were removed at each observation.

### Zebrafish Xenograft and M13_GD2_CF594 Targeting

At 2 days post‐fertilization (dpf), embryos were manually dechorionated, anesthetized with 0.16 mg mL^−1^ tricaine, and positioned in plastic grooves filled with 2% methylcellulose in PBS. NB cells were labeled for 20 min at 37 °C using Vybrant^TM^ DiI or Vybrant^TM^ DiO Cell‐Labeling Solution (5 µg mL^−1^, Thermo Fisher Scientific), a lipophilic dye that uniformly stains the cell membrane. After labeling, cells were suspended in 10 µL PBS or the appropriate culture medium at a concentration of 1 × 10^5^ cells µL^−1^, loaded into glass capillary needles, and microinjected into the yolk sac of each embryo (≈200 cells per embryo) using a WPI PicoPump device. To evaluate the in vivo tumor‐targeting specificity of M13_GD2_, zebrafish embryos were xenografted with Vybrant DiO‐labeled LAN‐5 (GD2‐positive) or Vybrant^TM^ DiO‐labeled SK‐N‐BE(2)C (GD2‐negative) NB cells. Following injection, embryos were incubated at 33 °C and monitored daily until 3 days post‐injection (dpi), the experimental endpoint. Dead embryos were removed at each observation. At 1 dpi, embryos were incubated overnight with M13_GD2_CF594 (2 µm CF59 equivalent to 6 nm M13_GD2_) in fish water at 33 °C under dark conditions to allow for phage internalization and binding. At 2 dpi, embryos were thoroughly washed with fresh water to remove unbound phages and maintained in fish water. Confocal fluorescence imaging was performed at 3 dpi following anesthesia with 0.16 mg mL^−1^ tricaine and immobilization in 1.0% low‐melting agarose. Images were acquired using a Nikon Eclipse Ti2 inverted microscope equipped with a confocal scanning unit and a 20x objective. M13_GD2_CF594 was excited at 561 nm and detected at ≈615–640 nm (Texas Red); Vybrant^TM^ DiO was excited at 488 nm and detected at 500–550 nm. Images were processed using Fiji (ImageJ), and colocalization was quantified by Pearson's correlation coefficient to assess the selective binding of the phage to GD2‐positive tumor xenografts.

### Zebrafish Xenograft and M13_GD2_RB Mediated PDT

At 1 dpi, xenografted embryos with DiO‐labeled LAN‐5 (or SK‐N‐BE(2)C NB cells were incubated overnight at 33 °C in the dark with 1 µm RB‐conjugated M13_GD2_ phage (M13_GD2_RB) in fish water to allow internalization and tumor‐specific binding. At 2 dpi, embryos were thoroughly washed with fresh water to remove unbound phages and maintained overnight in fresh fish water under dark conditions. At 3 dpi, targeted photoactivation was performed by exposing embryos to a white LED light source (Valex cold white LED, 24 mW cm^−^^2^ irradiance) positioned 30 cm above the plate for 10 min at room temperature, while control embryos were kept in dark. Immediately after irradiation, embryos were transferred to fresh fish water, anesthetized with 0.016% tricaine, and immobilized in 1.0% low‐melting‐point agarose in 35 mm glass‐bottom dishes for 6 h. Confocal Z‐stack imaging was performed at 0 h post‐irradiation using an Olympus IX83 P2ZF with Yokogawa CSU‐W1 spinning disk equipped with a 20× objective. Embryos were reimaged 6 h post‐irradiation under identical settings to evaluate time‐dependent phototoxic effects. DiO was excited at 488 nm and detected at 500–550 nm.

### Annexin V‐FITC Assay Post‐PDT in Zebrafish Xenograft

Xenograft and M13_GD2_RB‐mediated PDT was performed as described in the Materials and Methods section. Six hours post‐irradiation, embryos were enzymatically dissociated using a digestion solution containing PBS 1X, 0.25% phenol red–free trypsin, 1 mm EDTA, and 2.2 mg mL^−1^ Collagenase P. Enzymatic digestion was stopped with 1 mm CaCl_2_ and 10% fetal calf serum. Single‐cell suspensions were filtered through a 40 µm mesh and stained with Annexin V‐FITC (5 µL) in annexin binding buffer (10 mm HEPES, 140 mm NaCl, 2.5 mm CaCl_2_, pH 7.4) for 15 min at room temperature. Cells were then washed and immediately analyzed by flow cytometry. Gating strategies focused on identifying DiI‐positive tumor cells and the Annexin V‐FITC‐positive cell among them. A minimum of 50 000 events per sample were acquired using a Bio‐Rad S3e Cell Sorter and analyzed with FlowJo software.

### Imaging and Data Analysis of Zebrafish Embryos

High‐resolution imaging of embryos at 5 dpf was performed using a Nikon Eclipse Ti2 inverted confocal microscope and Olympus IX83 P2ZF with Yokogawa CSU‐W1 spinning disk. Quantification of fluorescent signals within defined regions of interest (ROIs) was carried out using the open‐source ImageJ/Fiji software, following previously published methods.^[^ ^72^ ^]^ A threshold was set to distinguish signal‐positive pixels from background, with all pixels below this cutoff excluded from analysis. Fluorescence intensities were normalized and expressed as arbitrary units (AU) to allow comparison across experiments. Imaging acquisition parameters, including laser excitation power and detector settings, were kept consistent between experimental groups to ensure comparability.

### Statistical Analysis

Statistical analyses were conducted using GraphPad Prism version 8.2.1. All datasets were derived from independent biological replicates. For cell culture experiments, each biological repeat was performed using a freshly thawed aliquot from the original cell stock to ensure consistency. Sample sizes in zebrafish embryos were obtained after collection and randomization from multiple mating events, excluding unfertilized eggs or embryos displaying very early developmental defects in all conditions. Data distribution was assessed using the Shapiro–Wilk test for normality (*α* = 0.05). If the data followed a normal distribution, parametric tests, such as the unpaired *t*‐test or one‐way ANOVA (for comparisons across multiple groups), were applied. In those cases, when data did not meet normality assumptions, nonparametric alternatives were used, specifically the Mann–Whitney U‐test or the Kruskal–Wallis test for multiple group comparisons. To adjust for multiple comparisons, Sidak's multiple comparison test or Tukey's test was applied following ANOVA, and Dunnett's test was used following Kruskal–Wallis analysis, as appropriate. The specific statistical tests used for each experiment are detailed in the corresponding figure legends. Sample sizes are also reported in the figure legends and represent the number of animals (for in vivo studies) or experimental units (for in vitro studies). Data are expressed as means with individual data points, and error bars indicate either the standard error of the mean (SEM) or standard deviation (SD), as specified.

## Conflict of Interest

The authors declare no conflict of interest.

## Author Contributions

S.K.Z. contributed to investigation, experiments, formal analysis, data curation, methodology, visualized and conceptualized the project, and wrote the initial draft. N.F., P.D.R., R.S., P.E.C., L.U., M.N., A.P., L.P., S.A., G.M., Z.A.D., M.D.S., A.R., L.F., M.S., L.C., G.Z., M.Z., M.D.G., F.D.M., RB, and E.V. contributed to investigation, data curation, and methodology formal analysis. M.C., A.D, and G.P. performed formal analyses, data interpretation, methodology, conceptualized and supervised the project, acquired funding, wrote and revised the manuscript.

## Supporting information



Supporting Information

## Data Availability

The data that support the findings of this study are available in the supplementary material of this article.
