# Supplementary material for: Systematic Targeting of GD2‐Positive Neuroblastoma Tumors With a Photooncolytic Phage Nanovector Platform
Source: Adv Sci (Weinh). 2025 Aug 13;12(38):e15356. doi: 10.1002/advs.202415356 (PMC12520574; doi:10.1002/advs.202415356)
Supplement: Supplementary file 1 — Supporting Information [file ADVS-12-e15356-s001.docx]

**Systematic Targeting of GD2-positive Neuroblastoma Tumors with a Photooncolytic Phage Nanovector Platform**

Suleman Khan Zadran^1,4,5^, Nicola Facchinello^1,6^, Piergiuseppe De Rosa^1^, Roberto Saporetti^2^, Paolo Emidio Costantini^1,4^, Luca Ulfo^1^, Michela Nigro^1,4^, Annapaola Petrosino^1^, Lucia Pappagallo^1^, Sara Aloisi^1^, Giorgio Milazzo^1^, Zainul Abe Din^1^, Alberto Rigamonti^1^, Leonardo Flora^1^, Martina Santulli^1^, Leonardo Cimadom^1^, Giampaolo Zuccheri^1^, Mattia Zangoli^3^, Manuele Di Sante^2^, Matteo Di Giosia^2,4^, Francesca Di Maria^3^, Roberto Bernardoni^1^, Eveline Barbieri^5^, Matteo Calvaresi^2,4§^, Alberto Danielli^1,4,§^, Giovanni Perini^1,4§^

1 Department of Pharmacy and Biotechnology Alma Mater Studiorum – University of Bologna, via Francesco Selmi 3, 40126 Bologna, Italy

2 Department of Chemistry “Giacomo Ciamician”, Alma Mater Studiorum – University of Bologna, Via Francesco Selmi 2, 40126 Bologna, Italy

3 Institute for Organic Synthesis and Photoreactivity (ISOF), National Research Council of Italy (CNR), Via P. Gobetti 101, Bologna, I-40129 Italy

4 Istituto di Ricerca e Cura a Carattere Scientifico (IRCCS) AOUBO Sant Orsola – Laboratory of Preclinical and Translational Research in Oncology (PRO), Bologna, Italy

5 Department of Pediatrics, Section of Hematology-Oncology, Texas Children's Cancer and Hematology Centers, Baylor College of Medicine, Houston, TX, 77030, USA.

6 Istituto di Ricerca e Cura a Carattere Scientifico (IRCCS) Istituto delle Scienze Neurologiche di Bologna, Programma di Neurogenetica, Bologna, Italy.

§ to whom correspondence should be addressed : M.C., [matteo.calvaresi3@unibo.it](mailto:matteo.calvaresi3@unibo.it); A.D., [alberto.danielli@unibo.it](mailto:alberto.danielli@unibo.it); G.P., [giovanni.perini@unibo.it](mailto:giovanni.perini@unibo.it)

**Supplementary Information and Figures**

**Supplementary Figure S1.**

**Supplementary Figure S1**. **Validation of ST8SIA1 expression (mRNA and protein) in NB cells and specificity of M13_GD2_ targeting in mixed cell populations**. **A)** ST8SIA1 mRNA expression in GD2-positive cell lines (LAN-5, CHP-134, IMR-32, Kelly, SH-SY5Y) showed high ST8SIA1 expression. In contrast, the GD2-negative cell line SK-N-AS expressed low ST8SIA1 transcript levels, while SK-N-SH and SK-N-BE(2)C demonstrated minimal expression. **B)** Western blot analysis of ST8SIA1 (GD3 synthase, ~43 kDa) expression in NB cell lines. GD3 synthase expression was detected in GD2-positive NB cell lines (LAN-5, CHP-134, IMR-32, Kelly, and SH-SY5Y) using an anti-GD3 synthase antibody. No or weak detectable signal was observed in GD2-negative NB lines (SK-N-AS, SK-N-SH, SK-N-BE(2)C). **C)** Immunofluorescence validation of M13_GD2_ phage targeting specificity using a co-culture model. HEK293 (GD2-negative) and LAN-5 (GD2-positive) cells were seeded on a single coverslip in a spatially organized manner (left: HEK 293; middle: 1:1 mix; right: LAN-5). Cells were stained with M13_GD2_ and anti-GD2 antibodies, followed by FITC-conjugated anti-M13 pVIII (magenta) and Cy3-conjugated anti-mouse IgG (green). Hoechst was used for nuclear staining (blue). No GD2 and M13_GD2_ signal was observed in HEK 293 cells (left), while strong signals for GD2 and M13_GD2_ were observed in LAN-5 cells (right). In the middle region, approximately 50% of cells (white circles: LAN-5) were positive for both GD2 and M13_GD2_, whereas the remaining (orange circles: HEK293) showed no signal, demonstrating M13_GD2_ specificity toward GD2-expressing NB cells. All images were acquired by a 40X, NIKON Eclipse Ti2/A1R confocal microscope. All scale bars represent 10 µm.

Supplementary Figure S2.

**Supplementary Figure S2**. **Optimization of M13_GD2_ phage concentration and validation of conjugated phage with CF™488A bioconjugates**. **A)** M13_GD2_ concentration optimization using various concentrations of phage (0.016, 0.166, 1.66 nM) tested on IMR-32 and Kelly (GD2-positive) cell lines and SK-N-SH (GD2-negative) cell line, immunostaining: pVIII of M13_GD2_ (Cy3: magenta) nuclei (Hoechst: cyan). **B)** MFI difference of M13_GD2_ and M13_WT_ on panels of NB cell lines, indicating strong binding by M13_GD2_ to GD2-positive cells, while no or minimal binding by M13_GD2_ or M13_WT_ to GD2-negative cells. **C)** Flow cytometry analysis validation of M13_GD2_ conjugated with CF488 on panels of NB cell lines.

Supplementary Figure S3.

**Supplementary Figure S3. Intracellular ROS production and Annexin-V-FITC staining**. **A)** Photodynamic-induced intracellular ROS was determined by H_2_DCFDA probe with GD2-positive (IMR-32 and Kelly) and GD2-negative (SK-N-AS and SK-N-BE(2)C) cell lines. Data are presented as the mean ± SD of 3 experiments. **B)** PI and Annexin-V-FITC staining were performed to assess M13_GD2_RB-mediated effects 24 hours post-PDT under light (irradiated) and dark (non-irradiated) conditions. Statistical significance was calculated by one-way ANOVA followed by Dunn's multiple comparisons test. The significance of differences is indicated as ns (not significant, p>0.05), * (p<0.05), **(p<0.01), ***(p<0.001), **** (p<0.0001).

**Supplementary Figure S4.**

**Supplementary Figure S4. A)** Schematic representation of Kelly transduced with Renilla luciferase and SK-N-AS with firefly luciferase, followed by co-culturing, phage incubation and PDT. **B)** Survival rates were determined by dual luciferase assay of mixed population cells, irradiated with white LED light 24 hours post-PDT treatment with M13_GD2_RB; controls were kept in the dark. Data are presented as the mean ± SD of 3 experiments. The significance of differences is indicated as ns (not significant, p>0.05), * (p<0.05), **(p<0.01), **** (p<0.0001).

**Supplementary Figure S5.**

**Supplementary Figure S5**: **Intracellular ROS production and intrinsic fluorescence of TD-conjugated M13_GD2_ virions**. **A)** Photodynamic-induced intracellular ROS determined by H_2_DCFDA probe with GD2-positive (IMR-32 and Kelly) and GD2-negative (SK-N-AS and SK-N-SH) cell lines. Data are presented as the mean ± SD of 3 experiments. **B)** M13_GD2_ conjugated with TD tested on IMR-32 and Kelly (GD2-positive) and SK-N-SH (GD2-negative), TD was excited (an excitation wavelength of 561 nm and an emission wavelength of 550−650 nm), all images were acquired by 40X, NIKON Eclipse Ti2/AIR confocal microscope. Statistical significance was calculated by one-way ANOVA followed by Dunn's multiple comparisons test. The significance of differences is indicated as ns (not significant, p>0.05), * (p<0.05), **(p<0.01), ***(p<0.001), **** (p<0.0001).
